# Ultrasound Trigger Ce‐Based MOF Nanoenzyme For Efficient Thrombolytic Therapy

**DOI:** 10.1002/advs.202304441

**Published:** 2024-04-04

**Authors:** Jianggui Shan, Ling Du, Xingang Wang, Sidi Zhang, Yiping Li, Song Xue, Qianyun Tang, Peifeng Liu

**Affiliations:** ^1^ Department of Cardiovascular Surgery Reiji Hospital Shanghai Jiao Tong University School of Medicine Shanghai 200127 China; ^2^ State Key Laboratory of Systems Medicine for Cancer Shanghai Cancer Institute Renji Hospital School of Medicine Shanghai Jiao Tong University Shanghai 200032 China; ^3^ Shanghai University of Traditional Chinese Medicine Shanghai 201203 China

**Keywords:** mesenchymal stem cell membrane, metal–organic framework, nanozyme, reactive oxygen species, thrombolysis

## Abstract

The inflammatory damage caused by thrombus formation and dissolution can increase the risk of thrombotic complications on top of cell death and organ dysfunction caused by thrombus itself. Therefore, a rapid and precise thrombolytic therapy strategy is in urgent need to effectively dissolve thrombus and resist oxidation simultaneously. In this study, Ce‐UiO‐66, a cerium‐based metal–organic framework (Ce‐MOF) with reactive oxygen species (ROS) scavenging properties, encapsulated by low‐immunogenic mesenchymal stem cell membrane with inflammation‐targeting properties, is used to construct a targeted nanomedicine Ce‐UiO‐CM. Ce‐UiO‐CM is applied in combination with external ultrasound stimulation for thrombolytic therapy in rat femoral artery. Ce‐UiO‐66 has abundant Ce (III)/Ce (IV) coupling sites that react with hydrogen peroxide (H_2_O_2_) to produce oxygen, exhibiting catalase (CAT) activity. The multi‐cavity structure of Ce‐UiO‐66 can generate electron holes, and its pore channels can act as micro‐reactors to further enhance its ROS scavenging capacity. Additionally, the porous structure of Ce‐UiO‐66 and the oxygen produced by its reaction with H_2_O_2_ may enhance the cavitation effects of ultrasound, thereby improving thrombolysis efficacy.

## Graphical abstract

An inflammation‐targeting nanodrug, Ce‐MOF‐CM, prepared by coating reactive oxygen species scavenging Ce‐UiO‐66 with low‐immunogenic mesenchymal stem cell membranes, is applied in combination with external ultrasound stimulation for thrombolytic therapy in rat femoral artery. The porous structure of Ce‐UiO‐66 and the oxygen produced by its reaction with H_2_O_2_ may enhance the cavitation effects of ultrasound, thereby improving thrombolysis efficacy.

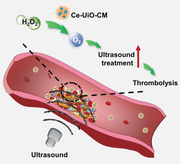

## 1 Introduction

Cardiovascular and cerebrovascular diseases are among the leading causes of death worldwide.^[^ ^1^ ^]^ One of the main factors that contribute to these diseases is intravascular thrombosis,^[^ ^2^ ^]^ which blocks blood flow and can result in myocardial infarction, ischemic stroke, and pulmonary embolism.^[^ ^3^ ^]^ Current treatments for thrombosis include antiplatelet therapy, anticoagulation therapy, thrombolytic drug therapy, and surgical treatment.^[^ ^4^ ^]^ However, each of these treatments has its limitations and drawbacks. For example, surgical intervention is effective but invasive, costly, and risky. Drug therapies are in rapid development, but they often cause bleeding complications, have short half‐lives, and lack specificity.^[^ ^5^ ^]^ Moreover, the abnormal vascular microenvironment after thrombosis formation is one of the major risks for inducing thrombus reformation. Thrombus formation and dissolution can trigger inflammatory damage and generate a large amount of reactive oxygen species (ROS),^[^ ^6^ ^]^ significantly increasing the overall oxidative stress level and lowering the local pH in the thrombus microenvironment.^[^ ^7^ ^]^ ROS generated by the injured endothelium and activated platelets at the thrombus site in turn lead to further endothelial dysfunction and platelet activation, thereby facilitating the propagation of thrombus.^[^ ^8^ ^]^ ROS also mediates endothelial expression of inflammatory cytokines and promotes platelet–endothelium interactions and vessel occlusion.^[^ ^9^ ^]^ Therefore, excessive ROS further worsens vascular conditions and increases the risk of thrombotic complications by inducing platelet aggregation and stimulating the overexpression of inflammatory cytokines in endothelial cells.^[^ ^10^ ^]^ The ROS level of the thrombus site is about twice that of normal blood vessels.^[^ ^11^ ^]^ Therefore, ROS‐scavenging in the thrombus microenvironment is key to improving the therapeutic effect of thrombolytic therapies.

Antioxidant enzymes such as catalase (CAT) are biocatalysts that can remove ROS under physiological and pathological conditions. However, the poor stability and high cost of natural antioxidant enzymes have limited their further practical applications.^[^ ^12^ ^]^ Nanoenzymes have attracted widespread attention by overcoming the drawbacks of natural antioxidant enzymes by combining the advantages of both nanomaterials and natural enzymes. Given their excellent catalytic performance, good stability, and ease of modification, most nanoenzymes still face challenges such as the small number of exposed active sites, the lack of the multilevel structure of natural enzymes, and the tendency toward self‐aggregation.^[^ ^13^ ^]^ In addition, most of the nanoenzymes only utilize surface atoms for enzyme‐like catalysis, while many internal atoms are either inactive or cause unwanted side reactions. Among all the nanoenzymes, metal–organic frameworks (MOFs) and their derivatives stand out as they have well‐defined coordination networks, mesoporous structures, and tunable pore size distribution.^[^ ^14^ ^]^ The controllable cavities and channels in their structures can provide hydrophobic coordination environments similar to those in natural enzymes, making them one of the most promising nanoenzymes.^[^ ^15^ ^]^ The diversity and biocompatibility of metal ions and ligands in MOFs also allow their direct application as therapeutic agents.^[^ ^16^ ^]^ Cerium, a transition metal element with multiple valences, can be used to construct nano‐sized Ce‐MOFs. The reversible conversion of Ce (III) and Ce (IV) oxidation–reduction pairs can be exploited to selectively clear ROS under physiological conditions.^[^ ^17^ ^]^

To overcome the problems such as immune recognition and clearance, and nonspecific interactions in vivo, cell membrane coating technology has emerged as a promising strategy to endow MOFs with enhanced biocompatibility, stability, and targeting ability.^[^ ^18^ ^]^ Among various cell membranes, mesenchymal stem cell (MSC) membranes are particularly attractive because MSCs can aggregate at inflammatory sites and recognize target cells by the target biomolecules on their membrane through cell–cell interaction and chemokines.^[^ ^19^ ^]^ The CD18 protein on the membrane of MSC can specifically bind to intercellular adhesion molecule‐1 (ICAM‐1),^[^ ^19^, ^20^ ^]^ which is overexpressed on vascular endothelium in the thrombosis area.^[^ ^21^ ^]^ Therefore, MSC membrane‐coated MOFs can act as efficient nanoenzymes that can actively and effectively target thrombus formation sites.

**Figure 1:**
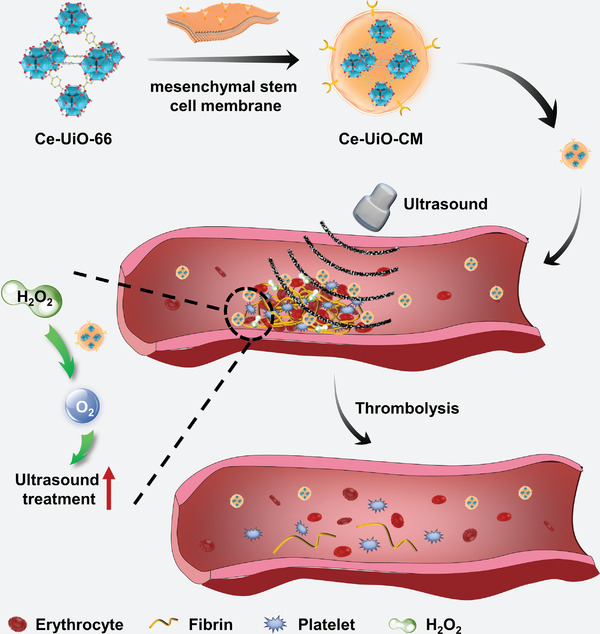
Schematic illustration of the synthesis of Ce‐UiO‐CM and its application in thrombolytic therapy in combination with ultrasound.

In this study, we developed an inflammation‐targeting nanodrug, Ce‐MOF‐CM, by coating ROS‐scavenging Ce‐MOFs (Ce‐UiO‐66) with low‐immunogenicity MSC membranes (**Figure** **1**). The coexistence of Ce (III) and Ce (IV) in the Ce‐MOFs gave it the CAT activity to allow its reaction with hydrogen peroxide (H_2_O_2_) to produce oxygen. Moreover, the ROS scavenging ability of Ce‐UiO‐66 can be further enhanced with its multi‐porous structure generating electron holes and the channels acting as micro‐reactors. We used photoacoustic imaging to show that Ce‐MOF‐CM effectively increased blood oxygen saturation at the thrombotic site by reacting with H_2_O_2_. We also combined Ce‐UiO‐CM with external ultrasound stimulation for femoral artery thrombolytic therapy and observed excellent thrombolytic effects both in vitro and in vivo, which may be attributed to the porous structure and oxygen production of Ce‐UiO‐66 that can amplify the cavitation effect of ultrasound.

## 2 Results

### 2.1 Preparation and Characterization of Ce‐UiO‐66

**Figure 2:**
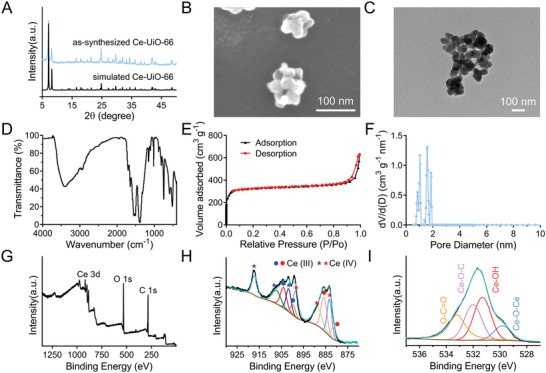
Characterization of Ce‐UiO‐66. A) The powder X‐ray diffraction (XRD) patterns of simulated Ce‐UiO‐66 and as‐synthesized Ce‐UiO‐66. B) The scanning electron microscopy (SEM) image of Ce‐UiO‐66. The scale bar is 100 nm. C) The transmission electron microscopy (TEM) image of Ce‐UiO‐66. The scale bar is 100 nm. D) The Fourier‐transform infrared spectroscopy (FTIR) spectra of Ce‐UiO‐66. E) The N_2_ sorption isotherm of Ce‐UiO‐66. F) The corresponding pore size distribution of Ce‐UiO‐66 was calculated by the DFT method. G) The XPS spectra of Ce‐UiO‐66. H) The magnified Ce 3d XPS spectrum of Ce‐UiO‐66. I) The magnified O 1s XPS spectrum of Ce‐UiO‐66.

Ce‐UiO‐66 has catalytic and photocatalytic properties due to the presence of Ce(III)/Ce(IV) redox couples, allowing for ROS scavenging with its strong antioxidant activity.^[^ ^22^ ^]^ It can withstand extreme pH values and temperatures and can be adopted for a wide range of working conditions. Ce‐UiO‐66 was successfully prepared by solvothermal method using ammonium cerium (IV) nitrate as the metal ion source and 1,4‐dicarboxybenzene (BDC) as the organic linker.^[^ ^23^ ^]^ The structure of Ce‐UiO‐66 was confirmed by X‐ray diffraction (XRD) analysis, which showed characteristic peaks at 2θ values of 7.1°, 8.2°, 11.6°, 13.6°, and 14.3° corresponding to the (111), (200), (220), (311), and (222) planes, respectively (**Figure** **2**A).^[^ ^24^ ^]^ Scanning electron microscopy (SEM) and transmission electron microscopy (TEM) imaging revealed that Ce‐UiO‐66 consisted of mostly spherical nanoparticles with an average size of 20–50 nm (Figure 2B,C). The high‐resolution TEM (HRTEM) image (Figure S1A, Supporting Information) and corresponding selected area electron diffraction (SAED) patterns (Figure S1B, Supporting Information) were consistent with the XRD results and demonstrated the successful synthesis of Ce‐UiO‐66. The energy‐dispersive X‐ray (EDX) spectroscopy elemental mapping results (Figure S1C, Supporting Information) showed that Ce, C, and O elements were homogeneously distributed in the particles of Ce‐UiO‐66, further verified that Ce‐UiO‐66 was successfully prepared. Solution ^1^H and ^13^C nuclear magnetic resonance (NMR) spectra (Figure S2, Supporting Information) were used to confirm the incorporation of the BDC linker. The Fourier‐transform infrared (FTIR) spectrum of Ce‐UiO‐66 (Figure 2D) showed clear stretching vibrations of O─H (3400 cm^−1^), carboxyl groups (1545 cm^−1^ for asymmetric and 1385 cm^−1^ for symmetric), and Ce─O bonds (515 cm^−1^).^[^ ^25^ ^]^ The specific surface area of Ce‐UiO‐66 was determined to be ≈1242 m^2^ g^−1^ by the Brunauer–Emmett–Teller (BET) method (Figure 2E), and the existence of micropores with sizes of 0.8, 1.0, 1.6, and 1.8 nm were confirmed by density functional theory (DFT) method (Figure 2F).

The surface composition of Ce‐UiO‐66 was investigated by X‐ray photoelectron spectroscopy (XPS) (Figure 2G–I). The Ce 3d XPS spectrum of Ce‐UiO‐66 revealed the existence of Ce ions in different valence states (Figure 2H), with six peaks corresponding to the spin–orbit splitting of Ce(IV) 3d_5/2_ (red asterisk) and 3d_3/2_ (blue asterisk) orbitals, and four peaks corresponding to the spin–orbit splitting of Ce(III) 3d_5/2_ (red circle) and 3d_3/2_ (blue circle) orbitals.^[^ ^26^ ^]^ The binding energies of the Ce(IV) 3d_3/2_ and Ce(IV) 3d_5/2_ were 917.0, 907.2, and 901.7 eV (blue asterisk), and 898.5, 888.6, and 883.2 eV (red asterisk) correspondingly. The binding energies of the Ce(III) 3d_3/2_ and Ce(III) 3d_5/2_ were 904.0 and 899.7 eV (blue circle), and 885.7 and 881.4 eV (red circle) correspondingly. The surface ratio of Ce(III) was calculated to be ≈35.0% (Table S1, Supporting Information), meaning that each Ce_6_O_4_(OH)_4_ cluster on the surface contained an average of 2 Ce(III) and 4 Ce(IV) ions. The O 1s signal of Ce‐UiO‐66 displayed four distinct peaks (Figure 2I), which were assigned to different oxygen species of the Ce_6_ cluster and the BDC ligand: oxygen atoms in uncoordinated carboxyl groups of the BDC ligands (O─C = O, 533.2 eV), coordinated carboxylate group between the BDC and Ce_6_ cluster (Ce─O─C, 532.0 eV), unsaturated cerium hydroxyl active site (Ce─OH, 531.3 eV), and bridging oxide bond within Ce_6_ clusters (Ce─O─Ce, 529.8 eV). The O1s XPS spectrum showed that the peak attributed to unsaturated Ce─OH (531.3 eV) was relatively high (33.9%) among the four O1s signal peaks (Table S2, Supporting Information), indicating the presence of abundant Ce─OH active sites on the surface of Ce‐UiO‐66.

### 2.2 Preparation and Characterization of Ce‐UiO‐CM

**Figure 3:**
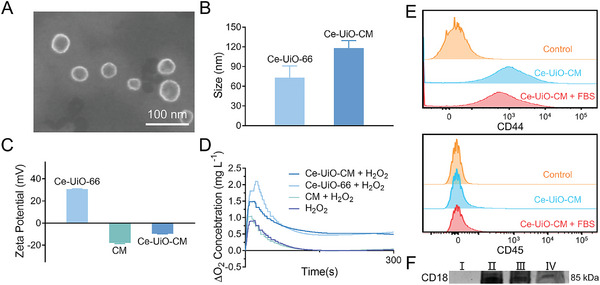
Characterization of Ce‐UiO‐CM. A) The SEM image of Ce‐UiO‐CM. The scale bar is 100 nm. B) The size distribution of Ce‐UiO‐CM and Ce‐UiO‐66 was measured by DLS analysis. Data are presented as mean ± standard error of the mean (SEM), *n* = 4. C) The surface charge of Ce‐UiO‐CM, CM, and Ce‐UiO‐66 is measured by zeta potential. Data are presented as mean ± standard error of the mean (SEM), *n* = 3. D) The O_2_ production by Ce‐UiO‐CM catalysis in aqueous solution. E) The expression of CD44 and CD 45 on Ce‐UiO‐CM with or without 10% fetal bovine serum (FBS) treatment as analyzed by flow cytometry. F) Western blot analysis of CD18 protein on Ce‐UiO‐66 (I), CM (II), Ce‐UiO‐CM (III), and Ce‐UiO‐CM treated with 10% FBS for 24 h (IV).

To improve the targeting ability of Ce‐UiO‐66 toward inflamed thrombus sites, mesenchymal stem cell membranes (CM) were coated to its surface. Ce‐UiO‐66 were wrapped with cell membrane fragments derived from rat bone marrow mesenchymal stem cells, a type of multipotent adult stem cell that can home to inflammatory sites, under the assistance of ultrasound to form the cell membrane‐camouflaged Ce‐UiO‐66 (Ce‐UiO‐CM). The resulting Ce‐UiO‐CM were spherical, with slightly increased nanoparticle size as seen in SEM images (**Figure** **3**A) compared to Ce‐UiO‐66. It had better dispersibility than Ce‐UiO‐66 due to the complete coverage of the particle surface by the merging of the adjacent protein–lipid patches (Figure S3, Supporting Information). The hydrated particle size of Ce‐UiO‐CM was ≈118 nm, which was 45 nm larger than that of the bare Ce‐UiO‐66 as revealed by dynamic light scattering (DLS) measurement (Figure 3B). The corresponding ζ‐potential measurement (Figure 3C) showed that the surface charge of Ce‐UiO‐CM changed to negative after CM coating, verifying the successful coating of the negatively charged CM on the Ce‐UiO‐66 surface. The presence of cell membrane proteins, which are critical for targeted delivery and therapy, on Ce‐UiO‐CM was also verified using the bicinchoninic acid (BCA) protein assay. The concentration of CM proteins coated on the surface of 0.1 mg mL^−1^ Ce‐UiO‐66 was determined to be 0.03 mg mL^−1^.

Thrombus formation is often accompanied by oxidative stress and inflammation, which lower the local pH and increase the risk of thrombosis. H_2_O_2_ is a major reactive ROS that is relatively stable compared to other ROS such as superoxide radicals (•$O_{2}^{-}$) and hydroxyl radicals (•OH) that have shorter half‐lives.^[^ ^27^ ^]^ H_2_O_2_ plays a crucial role in platelet activation and recruitment, so reducing H_2_O_2_ levels can inhibit these processes. Ce^3+^/Ce^4+^ electron pairs have been reported to produce oxygen by reacting with H_2_O_2_,^[^ ^28^ ^]^ as shown by the following equation:

(1) $$H_{2}O_{2}+2Ce^{4+}\to O_{2}+2H^{+}+2Ce^{3+}$$

(2) $$H_{2}O_{2}+2Ce^{3+}+2H^{+}\to 2H_{2}O+2Ce^{4+}$$

We detected the presence of Ce^3+^/Ce^4+^ electron pairs in Ce‐UiO‐66 and speculated that Ce‐UiO‐66 also has catalase (CAT) activity to break H_2_O_2_ into water and oxygen. To test this hypothesis, the oxygen concentration in the reaction system of Ce‐UiO‐CM and H_2_O_2_ was measured. The result showed that a significant increase in oxygen concentration was detected in the Ce‐UiO‐66 + H_2_O_2_ and Ce‐UiO‐CM + H_2_O_2_ group, while no apparent production of oxygen was observed in the pure H_2_O_2_ or CM + H_2_O_2_ group (Figure 3D), indicating that Ce‐UiO‐66 has CAT‐like activity while CM cannot generate oxygen by eliminating H_2_O_2_. The similar oxygen production between the Ce‐UiO‐66 + H_2_O_2_ and Ce‐UiO‐CM + H_2_O_2_ group indicates that the encapsulation of CM has no effect on the ability of Ce‐UiO‐CM to eliminate ROS.

The flow cytometry was used to analyze the expression of CD44 and CD 45 in Ce‐UiO‐CM. Figure 3E showed the existence of positive surface markers CD44 and the nonexistence of negative surface marker CD45 of MSC on Ce‐UiO‐CM,^[^ ^29^ ^]^ demonstrating the successful encapsulation of MSC membranes. Additionally, 10% fetal bovine serum (FBS) treatment had almost no effect on the expression of CD44 on the surface of Ce‐UiO‐CM (Figure 3E). As the CD18 protein can specifically bind to ICAM‐1 to achieve targeted delivery, the existence of the key membrane protein CD18 of cell membrane on Ce‐UiO‐CM was further identified by western blotting analysis. The immunoblotting confirmed that CD18 of Ce‐UiO‐CM was present and enriched, which could bestow the targeting properties on the NPs (Figure 3F). Additionally, the expression of CD18 protein (Figure 3F) in 10% FBS‐treated Ce‐UiO‐CM indicated that plasma proteins have almost no effect on the targeting ability of Ce‐UiO‐CM.

### 2.3 In Vitro Thrombolytic Efficacy of Ce‐UiO‐CM

**Figure 4:**
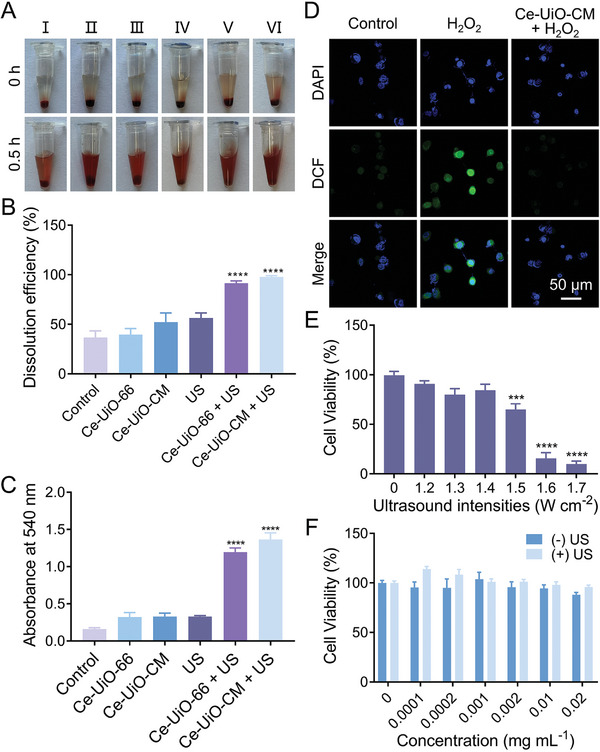
Assessment of the clot‐dissolution efficiency, CAT activity, and cytotoxicity of Ce‐UiO‐CM, Ce‐UiO‐66, and ultrasound treatment in vitro. A) Representative images of the blood clot preparations at 0 and 0.5 h after treatment with saline (I), Ce‐UiO‐66 (II), Ce‐UiO‐CM (III), ultrasound (IV), Ce‐UiO‐66 + US (V), and Ce‐UiO‐CM + US (VI). B) The quantified clot–dissolution efficiency of different treatment groups (corresponding to Figure 4A) in vitro. Data are presented as mean ± standard error of the mean (SEM), *n* = 4. ^****^ *p *< 0.0001, one‐way analysis of variance (ANOVA) with Tukey's multiple comparisons. C) The absorbance at 540 nm in different treatment groups (corresponding to Figure 4A) after 30 min of treatment. Data are presented as mean ± standard error of the mean (SEM), *n* = 4. ^****^ *p *< 0.0001, ANOVA with Tukey's multiple comparisons. D) The ROS removal capacity of Ce‐UiO‐CM in H_2_O_2_‐stimulated human umbilical vein endothelial cells (HUVECs). The scale bar is 50 µm. E) The cytotoxicity of ultrasound at different sound intensities in HUVECs. Data are presented as mean ± standard error of the mean (SEM), *n* = 4. ^***^ *p *< 0.001, ^****^ *p *< 0.0001, ANOVA with Tukey's multiple comparisons. F) The cytotoxicity of different concentrations of Ce‐UiO‐CM in HUVECs with or without ultrasound treatment. Data are presented as mean ± standard error of the mean (SEM), *n* = 4. ANOVA with Tukey's multiple comparisons. No obvious changes were observed.

Ultrasound is a high‐frequency mechanical wave that can cause cavitation as well as mechanical and thermal effects in tissues and thus can be utilized to improve the penetration of drugs into thrombotic tissues. In order to study the in vitro thrombolytic effect of Ce‐UiO‐CM under ultrasound stimulation, blood clot samples were treated with saline, Ce‐UiO‐66, Ce‐UiO‐CM, ultrasound (US), Ce‐UiO‐66 + US, or Ce‐UiO‐CM + US. All the treatments except saline caused the clots to shrink and release hemoglobin into the supernatant, turning the colorless supernatant into blood red, indicating clot dissolution (**Figure** **4**A). The mass loss of the clots was measured after 30 min of treatment to determine the efficiency of clot dissolution. The Ce‐UiO‐66 + US group and Ce‐UiO‐CM + US group showed significantly higher dissolution efficiency than that of the pure Ce‐UiO‐66, Ce‐UiO‐CM, and ultrasound group (Figure 4B), and the amount of hemoglobin released to the supernatant, as measured by light absorbance at 540 nm, during clot dissolution corresponded to the trend of the dissolution efficiency (Figure 4C). Notably, the thrombolysis efficiency of the Ce‐UiO‐CM + US group was slightly higher than that of the Ce‐UiO‐66 + US group, possibly because of the enhanced permeability brought about by CM under static and non‐blood flow conditions.

To investigate the cellular internalization mechanism, the HUVECs underwent pretreatment with ROS to induce endothelial cell activation and ICAM‐1 overexpression. After ROS treatment, a marked upregulation of ICAM‐1 on HUVECs was observed (Figure S4, Supporting Information). Moreover, intense green fluorescence from CD18 on Ce‐UiO‐CM was notably evident in HUVECs exhibiting high ICAM‐1 expression after 4 h of incubation with Ce‐UiO‐CM, whereas HUVECs with low ICAM‐1 expression only gave a faint fluorescence. These findings suggest enhanced cellular uptake of Ce‐UiO‐CM by HUVECs with elevated ICAM‐1 levels, highlighting the specificity of Ce‐UiO‐CM for targeting inflamed endothelial cells. The effect of Ce‐UiO‐CM on intracellular ROS was investigated in human umbilical vein endothelial cells (HUVECs) by confocal laser scanning microscopy (CLSM) using 2′,7′‐dichlorodihydrofluorescein diacetate (DCFH‐DA) as the ROS probe. The intracellular ROS (green fluorescence) level was significantly reduced by Ce‐UiO‐CM treatment compared to that of the H_2_O_2_‐stimulated HUVECs (Figure 4D), suggesting that Ce‐UiO‐CM could scavenge excessive ROS and protect endothelial cells from oxidative stress. To evaluate the safety of Ce‐UiO‐CM and ultrasound treatment for endothelial cells, the viability of HUVECs was assessed by cell counting Kit‐8 (CCK‐8) assay after treatment with ultrasound under different intensities or Ce‐UiO‐CM (or Ce‐UiO‐66) under different concentrations. 10 min of ultrasound treatment was applied to HUVECs, and the results showed that ultrasound intensities up to 1.4 W cm^−2^ did not significantly affect cell viability, while ultrasound intensity of 1.5 W cm^−2^ caused significant cell death (Figure 4E). Therefore, the ultrasound intensity of 1.4 W cm^−2^ was used for further experiments. Meanwhile, the CCK‐8 results showed that different concentrations of Ce‐UiO‐CM (Figure 4F) or Ce‐UiO‐66 (Figure S5, Supporting Information) had no adverse effect on the viability of HUVECs, regardless of ultrasound exposure, indicating that Ce‐UiO‐CM and ultrasound treatment are safe for endothelial cells. Altogether, the above in vitro results suggested that Ce‐UiO‐CM can enhance the thrombolytic effect of ultrasound and may have potential applications in thrombolytic therapy.

### 2.4 In Vivo Thrombolytic Therapy

**Figure 5:**
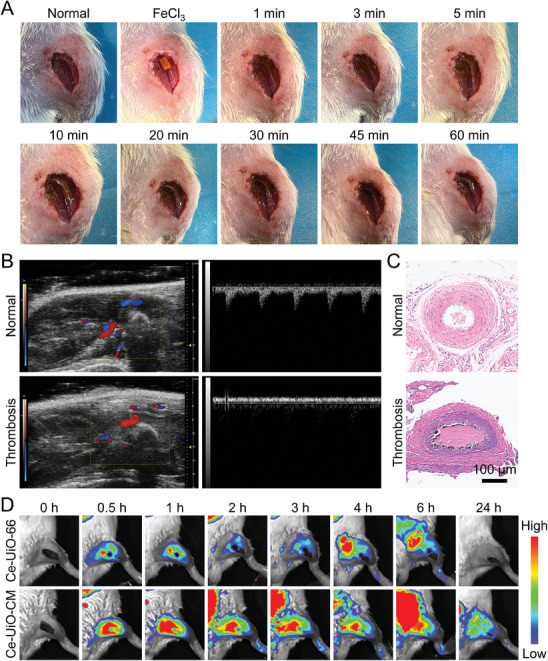
Establishment of the rat femoral artery thrombus model and thrombus targeting of Ce‐UiO‐CM in vivo. A) The establishment of rat femoral artery thrombosis model using the FeCl_3_ injury method. B) The changes of blood flow before and after thrombus formation in the rat femoral artery thrombosis model shown by Doppler ultrasound flow imaging. C) H&E staining images of blood vessels in the thrombus model and normal animal. D) The fluorescence distribution of ICG‐modified Ce‐UiO‐CM and Ce‐UiO‐66 in vivo. Fluorescence images were collected at 0, 0.5, 1, 2, 3, 4, 6, and 24 h after Ce‐UiO‐CM or Ce‐UiO‐66 injection through the tail vein.

To study the in vivo thrombus targeting of Ce‐UiO‐CM, a femoral artery thrombosis model was established in SD rats using the FeCl_3_ injury method (**Figure** **5**A).^[^ ^30^ ^]^ A piece of filter paper impregnated with FeCl_3_ solution was placed on the outer wall of the blood vessel for 1 min. Then, the filter paper was removed and the vessel was allowed to rest for ≈10 min until a visible thrombus formed. The blood flow before and after thrombus induction was monitored with Doppler ultrasound flow imaging. The results showed that the blood flow in the injured vessel was significantly reduced after thrombus formation (Figure 5B and Figure S6, Supporting Information). Hematoxylin–eosin (H&E) staining was performed to analyze the histological sections of the thrombosed vessel. A clear occlusion in the lumen of the thrombosed vessel was seen compared with the normal vessel (Figure 5C), confirming the successful establishment of the femoral artery thrombosis model. Additionally, the ICAM‐1 expression was increased on vascular endothelium in the thrombosis area compared with the normal vascular (Figure S7, Supporting Information). Pronounced green fluorescence emanating from CD18 on Ce‐UiO‐CM was predominantly localized in the vascular endothelium exhibiting ICAM‐1 overexpression. This localization indicates the specificity of Ce‐UiO‐CM for targeting inflammatory endothelial cells in the thrombotic area, underscoring its potential for directed therapeutic intervention in thrombosis.

To further study the thrombus targeting ability of our nanoenzyme in vivo, indocyanine green (ICG)‐modified Ce‐UiO‐66 or Ce‐UiO‐CM (Figure S8, Supporting Information) was injected into the animal intravenously and monitored by fluorescence imaging. Compared with the non‐targeting group (Ce‐UiO‐66), the fluorescence signal in the femoral artery region of the targeting group (Ce‐UiO‐CM) was significantly stronger, and the signal intensity reached its maximum at 0.5 h after injection for both groups (Figure 5D; Figure S9, Supporting Information). Moreover, the fluorescence signal in the non‐targeting group decayed rapidly, while that in the targeting group persisted for up to 2 h, demonstrating excellent targeting and retention of Ce‐UiO‐CM in the thrombus site (Figure S9B, Supporting Information). The distribution of Ce‐UiO‐CM (Figure S10, Supporting Information) in thrombus modeling femoral artery was consistent with the results in Figure 5D and Figure S9 (Supporting Information), confirming the targeting of Ce‐UiO‐CM to the thrombus. Moreover, Ce‐UiO‐66 and Ce‐UiO‐CM exhibited similar distribution characteristics (Figure S10, Supporting Information) in major organs (including the heart, liver, spleen, lung, and kidney). To study the pharmacokinetic performance of Ce‐UiO‐CM, four rats were injected with Ce‐UiO‐CM solution through tail vein injection. The plasma concentration of Ce measured by inductively coupled plasma mass spectrometry (ICP‐MS) showed that Ce‐UiO‐CM could almost exclusively clear after 72 h from the blood (Figure S11, Supporting Information). The main pharmacokinetic parameters were calculated by a noncompartment model and presented in Table S3 (Supporting Information). The terminal elimination half‐life (T_1/2_) was 20.25 ± 4.53 h, ensuring sufficient accumulation of Ce‐UiO‐CM.

**Figure 6:**
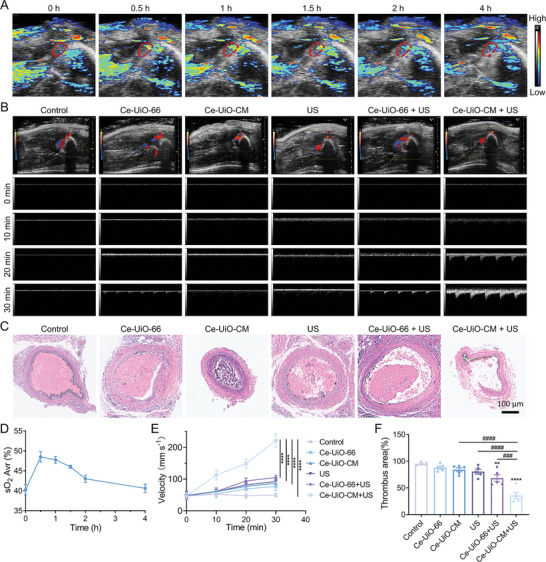
Evaluation of the oxygen production ability and the thrombolytic efficacy of Ce‐UiO‐CM in vivo. A) The photoacoustic signal, indicative of the blood oxygen saturation level, at the embolic site after Ce‐UiO‐CM administration. B) The changes in blood flow in the clotted femoral artery area in different treatment groups. C) The representative histological images of the femoral artery in different treatment groups. The scale bar is 100 µm. D) The blood oxygen saturation level at the embolic site after Ce‐UiO‐CM injection through the caudal vein. Data are presented as mean ± standard error of the mean (SEM), *n* = 4. E) The changes of blood flow in the femoral artery area as revealed by Doppler flow imaging in different treatment groups. Data are presented as mean ± standard error of the mean (SEM), *n* = 5. ^****^ *p *< 0.0001, ANOVA with Tukey's multiple comparison. F) The quantified thrombus area of the femoral artery in each treatment group. Data are presented as mean ± standard error of the mean (SEM), *n* = 5. ^**^ *p *< 0.01, ^###^ *p *< 0.001, ^####^, ^****^ *p *< 0.0001, ANOVA with Tukey's multiple comparison.

The in vivo oxygen production ability of Ce‐UiO‐CM was evaluated by measuring the blood oxygen saturation using photoacoustic imaging. The result showed that the blood oxygen saturation in the femoral artery thrombotic region increased significantly after intravenous injection of Ce‐UiO‐CM (**Figure** **6**A), reaching a peak at 0.5 h and remaining elevated for up to 2 h (Figure 6D). Correspondingly, after injection of Ce‐UiO‐66, the blood oxygen saturation in the femoral artery thrombotic region reached a peak at 0.5 h and then rapidly decreased (Figure S12, Supporting Information). This was consistent with the distribution of Ce‐UiO‐CM in the tissue as indicated by the fluorescence imaging data. Based on these results, ultrasound treatment was performed at 0.5 h after the tail vein injection of Ce‐UiO‐CM.

Microbubbles can significantly improve ultrasound echo intensity and enhance the contrast in ultrasound imaging.^[^ ^31^ ^]^ Upon the conversion of H_2_O_2_ to oxygen, there was a notable enhancement in ultrasound echo intensities in both the Ce‐UiO‐66 + H_2_O_2_ group and the Ce‐UiO‐CM + H_2_O_2_ group (Figure S13A,B, Supporting Information). Moreover, following the administration of Ce‐UiO‐66 and Ce‐UiO‐CM, a substantial increase in B‐mode ultrasound echo intensity was observed at the thrombus site (Figure S13C,D, Supporting Information). This enhancement can be attributed to the increased generation of oxygen from H_2_O_2_ decomposition catalyzed by the Ce‐based MOFs. This oxygen generation not only contributes to the improved echo intensity but also potentially aids in better visualization and localization of the thrombus during ultrasound imaging, thereby facilitating more precise and targeted therapeutic interventions. The changes in blood flow in the femoral artery area were analyzed using Doppler flow imaging (Figure 6B). The Ce‐UiO‐CM + US treatment group showed a significant improvement in blood flow compared to the Ce‐UiO‐CM, Ce‐UiO‐66, or US treatment alone (Figure 6E). Thrombolytic efficiencies were quantified by measuring the ratio of the cross‐sectional area of the blood clot to the artery in the histological images.^[^ ^31b^ ^]^ As expected, the Ce‐UiO‐CM + US group achieved the best thrombolytic effect, producing the smallest clot area (≈36%) (Figure 6C,F), while the proportions for the saline group, Ce‐UiO‐66 group, Ce‐UiO‐CM group, US group, and Ce‐UiO‐66 + US group were 95%, 88%, 84%, 80%, and 68%, respectively (Figure 6F). Ultrasound technology operates primarily through mechanical or cavitation effects, and to some extent, thermal effects. In this context, inertial cavitation plays a pivotal role. It involves substantial expansion followed by rapid collapse of microbubbles when exposed to ultrasound sonication, resulting in powerful jetting forces that directly mechanically disrupt the clot.^[^ ^32^ ^]^ The Ce‐based MOFs enhance this process by catalyzing the decomposition of H_2_O_2_ into oxygen, thereby amplifying the cavitation effects. This increased cavitation not only improves ultrasound imaging contrast,^[^ ^33^ ^]^ but also significantly boosts the efficacy of thrombus treatment. This synergy between the Ce‐UiO‐CM and ultrasound results in a more potent thrombolytic therapy.

The DHE staining was performed to investigate the impact of Ce‐UiO‐CM on the level of ROS in thrombosed vessels.^[^ ^6^ ^]^ The endothelium of the femoral artery in the control, CM, and US groups showed a high intensity of DHE fluorescence while the fluorescence intensities were significantly reduced after Ce‐UiO‐66 and Ce‐UiO‐CM treatment with or without US (Figure S14, Supporting Information), indicating the ROS scavenging capacity of Ce‐UiO‐66 and Ce‐UiO‐CM in vivo. The proliferation of vascular smooth muscle cells (VSMCs) and the increase of collagen concentration are beneficial in vascular injury areas.^[^ ^20^ ^]^ Furthermore, smooth muscle cell marker α‐smooth muscle actin (α‐SMA) staining assay showed that the number of VSMCs was significantly increased in the thrombotic area after treatment with Ce‐UiO‐66 and Ce‐UiO‐CM with or without US (Figure S15, Supporting Information). Consistent with this result, Masson's trichrome staining showed that the concentration of collagen was also greatly upregulated after Ce‐UiO‐66 and Ce‐UiO‐CM treatment with or without US (Figure S16, Supporting Information). These findings indicated that Ce‐UiO‐66 and Ce‐UiO‐CM treatment improved these damaged blood vessels in the thrombotic area.

To assess the biocompatibility of Ce‐UiO‐CM combined with the US in vivo, a routine blood test (Figure S17, Supporting Information) was performed, and serum biochemical parameters associated with the liver (Figure S18, Supporting Information), renal (Figure S19, Supporting Information) and cardiac (Figure S20, Supporting Information) functions were measured. No significant change was found between the Ce‐UiO‐CM + US treatment group and the control group. In addition, the histological images of the major organs (including heart, liver, spleen, lung, and kidney) of the thrombosed rats revealed no evident organ damage or inflammatory lesions in the Ce‐UiO‐CM + US treatment group (Figure S21, Supporting Information), confirming the biosafety of this treatment strategy.

## 3 Discussion

The current therapeutic approaches for thrombosis encompass surgical intervention, antiplatelet therapy, anticoagulant therapy, and thrombolytic drug therapy. Each of these methods, however, presents specific challenges, including invasiveness, bleeding complications, a brief half‐life, insufficient specificity, and the potential for inflammatory damage. To address these issues, this study introduces an innovative nanomedicine that specifically targets inflammation, used in conjunction with ultrasound to achieve rapid and precise thrombolytic therapy. This novel strategy is characterized by its ability to effectively dissolve thrombi and exhibit antioxidative properties. It operates through three principal mechanisms: efficient scavenging of ROS, targeted action on thrombi, and accelerated thrombolysis facilitated by ultrasound application.

To effectively eliminate ROS, we constructed a Ce‐based MOF (Ce‐UiO‐66) as a nanoenzyme for ROS removal. This Ce‐based MOF addresses the limitations of natural antioxidant enzymes, notably their poor stability and high cost. The reversible conversion between Ce (III) and Ce (IV) oxidation‐reduction pairs in the Ce‐based MOF enables selective ROS elimination under both physiological and pathological conditions (Figures 3, 4; Figure S14, Supporting Information). Cerium dioxide (CeO_2_), known for containing Ce(III) and Ce(IV) oxidation–reduction pairs, is already utilized in treating cancer, acute kidney injury, and ischemic stroke due to its potent antioxidative activity and capacity for repeated ROS scavenging.^[^ ^28^, ^34^ ^]^ Compared to solid CeO_2_, the Ce‐based MOF, with controlled cavities and channels, offers a hydrophobic coordination environment akin to that of natural enzymes. The Ce‐UiO‐66 synthesized in this study features micropores of 0.8, 1.0, 1.6, and 1.8 nm (Figure 2F). Its multi‐cavity structure allows the Ce‐UiO‐66 to generate electron vacancies and its channels to function as microreactors, thereby augmenting its ROS scavenging efficiency. Furthermore, the synthesis of Ce‐UiO‐66 in this study was achieved via a solvothermal method. This approach is more straightforward and controllable compared to the templating method employed for the preparation of mesoporous hollow cerium oxide upconversion nanoparticles (Ce‐UCNPs).^[^ ^28a^ ^]^

Enhanced delivery efficiency and specificity, achieved through targeted delivery, substantially reduce the required dosage and associated side effects. In this study, low‐immunogenic MSC membranes were employed to encapsulate the Ce‐based MOF. The CD18 protein present on the MSC membrane specifically binds to ICAM‐1, which is overexpressed on inflamed vascular endothelium. This mechanism directs the Ce‐UiO‐CM specifically to inflamed endothelial cells within the thrombus area (Figure 5D; Figures S4, S7, S9, and S10, Supporting Information). The specific interaction between CD18 and ICAM‐1 has been previously utilized to enhance the targeting efficacy of a nano‐delivery platform (AM@ZIF@NM) to plaque endothelial cells, effectively mitigating inflammation in atherosclerotic lesions and alleviating atherosclerosis.^[^ ^20^ ^]^ This study utilizes targeted nanomedicine combined with ultrasound for rapid thrombolysis. However, ultrasound alone can induce thermal effects during the propagation in tissues, which are nonspecific. High‐intensity ultrasound can damage healthy tissue and lead to undesired off‐target effects.^[^ ^35^ ^]^ Therefore, the incorporation of targeted nanomedicine in this study allows for the achievement of therapeutic effects using much lower ultrasound intensities. This approach addresses the current limitations of ultrasound therapy by minimizing potential thermal and mechanical damage to surrounding healthy tissues, thereby enhancing the safety and efficacy of thrombolytic treatment.

Nanobiomedicine has increasingly been integrated with various therapeutic modalities for thrombolysis. For instance, a platform incorporating near‐infrared (NIR) light‐responsive liposomal gold nanorods has been developed for protein delivery, facilitating non‐hemorrhagic photothermal‐assisted thrombolysis.^[^ ^36^ ^]^ However, this approach's therapeutic depth is constrained by the limited penetration depth of the laser, as biological tissues absorb light. Conversely, the ultrasound technique employed in this study presents distinct advantages, such as robust spatiotemporal targeting, deep tissue penetration, high biosafety, absence of electromagnetic radiation, non‐invasiveness, and portability.^[^ ^37^ ^]^ The plasma‐derived nanoclusters have also been designed for site‐specific multimodality photo/magnetic thrombus theragnostic, improving the penetration of nanomedicines into thrombi by magnetic guidance.^[^ ^38^ ^]^ However, the ROS generated by methylene blue in the nanoclusters during photodynamic therapy (PDT) may increase the risk of thrombotic complications by inducing platelet aggregation and upregulating inflammatory cytokines in endothelial cells. The nanoenzyme used in this study can effectively eliminate ROS while rapidly dissolving thrombi with ultrasound, reducing oxidative damage and thrombotic complications associated with ROS, thus enhancing the safety of the treatment (Figures S15–S21, Supporting Information). Additionally, ultrasound stimulation also improves the drug penetration into thrombi. The penetration of tPA in a porous and magnetic microbubble platform (MMB‐SiO2‐tPA) by ultrasound extended several hundred micrometers within the thrombi.^[^ ^31b^ ^]^ However, the size of this platform (≈5 µm), subjected it to rapid clearance by the reticuloendothelial system, and its hydrophobic surface properties impede circulation time, with tPA activity maintaining only a half‐life of 1 h in the presence of its inhibitors in vitro. In contrast, the MSC membranes coating in our study prolonged the nanomedicine's terminal elimination half‐life to 20.25 ± 4.53 h, thus ensuring sufficient accumulation of Ce‐UiO‐CM. Moreover, the thrombotic fragments post‐ultrasound pulse therapy predominantly become subcapillary in size, significantly reducing the risk of distal embolism following ultrasound thrombolysis.^[^ ^39^ ^]^ In recent years, nanosystems have been combined with ultrasound, where perfluorocarbon nanoparticles encapsulated in acoustically sensitive liposomes can undergo phase transition under ultrasound, generating nanobubbles helpful for ultrasound thrombolytic therapy.^[^ ^40^ ^]^ Unlike the exogenous gases used in this strategy, the Ce‐based MOF used in this study converted endogenous ROS into O_2_ (Figure 6A,D; Figure S12, Supporting Information), not only alleviating the hypoxic environment of the thrombus formation area but also reducing the risk of thrombotic complications caused by ROS. This, combined with the specific targeting brought by the MSC membrane coating, made the treatment safer. However, due to the limited availability of endogenous ROS, the O_2_ generated for ultrasound thrombolytic therapy is also limited. The ultrasound‐triggered phase transition technology can be combined with the nanoenzyme to increase bubble production, thereby enhancing the therapeutic effect.

## 4 Conclusion

This study reported a novel strategy for femoral artery thrombolytic therapy based on a cerium‐based MOF (Ce‐UiO‐66) encapsulated in the membrane of mesenchymal stem cells. MSCs are known for their low immunogenicity and inflammation‐targeting ability, the cell membrane of which can enhance the delivery of Ce‐UiO‐66 to the thrombotic site. The presence of abundant Ce(III)/Ce(IV) coupling sites in Ce‐UiO‐CM gave it CAT‐like activity for ROS scavenging and oxygen production by reacting with H_2_O_2_. The multi‐mesoporous structure of Ce‐UiO‐CM allowed the generation of electron holes that act as micro‐reactors to further enhance its ROS scavenging activity, which can effectively increase blood oxygen saturation at the thrombotic site. The porous structure and oxygen generation of Ce‐UiO‐66 could amplify the cavitation effect of external ultrasound stimulation, leading to efficient thrombolysis both in vitro and in vivo.

## 5 Experimental Section

### Preparation of Ce‐UiO‐66

Ce‐UiO‐66 was prepared according to previous reports.^[^ ^18^ ^]^ In brief, (NH_4_)_2_Ce(NO_3_)_6_ (1.17 g, 2.135 mmol) and acetic acid (122 µL) were dissolved in 4 mL of deionized water and heated to 60 °C for 30 min, followed by cooling in an ice‐water bath. BDC (0.355 g, 2.135 mmol) dissolved in 18.76 mL N,N‐dimethylformamide (DMF) was then added to the cerium solution. After heating at 80 °C for 30 min, the mixture was cooled to room temperature, and the resulting Ce‐UiO‐66 was collected by centrifugation at 20000×g for 10 min. The Ce‐UiO‐66 collected was washed three times with DMF. For sample activation, the obtained Ce‐UiO‐66 was soaked in 50 mL DMF for 6 h, followed by centrifugation and pellet collection two times. The synthesized Ce‐UiO‐66 was immersed in 30 mL ethanol at 60 °C overnight three times to remove any residual DMF. The solid was then washed twice with water and stored at 4 °C.

Additional experimental details and characterization can be found in the Supporting Information.

## Conflict of Interest

The authors declare no conflict of interest.

## Supporting information

Supporting Information

## Data Availability

Research data are not shared.
