# Correction to “Ultrasound Trigger Ce‐Based MOF Nanoenzyme For Efficient Thrombolytic Therapy”

**DOI:** 10.1002/advs.75005

**Published:** 2026-04-09

Shan J, Du L, Wang X, Zhang S, Li Y, Xue S, Tang Q, Liu P. Ultrasound Trigger Ce‐Based MOF Nanoenzyme For Efficient Thrombolytic Therapy. *Adv. Sci*. 2024; 11: 2304441.

The affiliation of the first author, Jianggui Shan, and the corresponding author, Song Xue, was incorrectly listed as “Reiji Hospital.” The correct affiliation should be: “Department of Cardiovascular Surgery, Renji Hospital, School of Medicine, Shanghai Jiao Tong University, Shanghai, China.”

We apologize for this error.

