# Supplementary material for: Ultrasound Trigger Ce‐Based MOF Nanoenzyme For Efficient Thrombolytic Therapy
Source: Adv Sci (Weinh). 2024 Apr 4;11(20):2304441. doi: 10.1002/advs.202304441 (PMC11132072; doi:10.1002/advs.202304441)
Supplement: Supplementary file 1 — Supporting Information [file ADVS-11-2304441-s001.pdf]

ADVANCED SCIENCE
Open Access

Supporting Information

for *Adv. Sci.*, DOI 10.1002/advs.202304441

Ultrasound Trigger Ce-Based MOF Nanoenzyme For Efficient Thrombolytic Therapy

Jianggui Shan, Ling Du, Xingang Wang, Sidi Zhang, Yiping Li, Song Xue, Qianyun Tang* and Peifeng Liu**

Supporting Information

Ultrasound Trigger Ce-based MOF Nanoenzyme For Efficient Thrombolytic Therapy

Jianggui Shan, Ling Du, Xingang Wang, Sidi Zhang, Yiping Li, Song Xue, Qianyun Tang* and Peifeng Liu**

J. Shan, S. Xue
Department of Cardiovascular Surgery, Reiji Hospital, Shanghai Jiao Tong University School of Medicine, Shanghai 200127, P. R. China

L. Du, X. Wang, S. Zhang, Y. Li, Q. Tang, P. Liu
State Key Laboratory of Systems Medicine for Cancer, Shanghai Cancer Institute, Renji Hospital, School of Medicine, Shanghai Jiao Tong University, Shanghai 200032, P. R. China

Y. Li
Shanghai University of Traditional Chinese Medicine, Shanghai 201203, P. R. China

E-mail: xuesong@renji.com; qytang@shsci.org; lpf@sjtu.edu.cn

Materials. Ammonium cerium(IV) nitrate ($(NH_4)_2Ce(NO_3)_6$) was purchased from Shanghai Macklin Biochemical Co., Ltd. 1,4-dicarboxybenzene (BDC) and indocyanine green (ICG) were purchased from Shanghai Aladdin Reagent Co., Ltd. Acetic acid, N,N-dimethylformamide (DMF) and ethanol were purchased from Shanghai Adamas Reagent Co., Ltd. Hydrogen peroxide (H_2O_2) was purchased from Sangon Biotech (Shanghai) Co., Ltd.

Preparation of ICG-modified Ce-UiO-66. ICG-modified Ce-UiO-66 was prepared based on the coordinate assembly of ICG with Ce6 clusters on Ce-UiO-66. ICG (0.1 mg, 0.129 μmol) was added into the Ce-UiO-66 aqueous dispersion (1.0 $mg \cdot mL^{-1}$, 1 mL), and the mixture was stirred for 2 hours at 4 °C in dark. The resulting ICG-modified Ce-UiO-66 was collected by centrifugation at 20,000×*g* for 10 min. The solid was washed with water and stored at 4 °C.

Nanoparticle Characterization. The structural information of Ce-UiO-66 in solution was obtained using powder X-ray diffraction (XRD) on a SmartLab X-ray diffractometer (Rigaku, Japan). The morphology of the prepared Ce-UiO-66 was observed by transmission electron microscopy (TEM) on a HT7700 transmission electron microscope (Hitachi, Japan), scanning electron microscopy (SEM) on a SU8230 scanning electron microscope (Hitachi, Japan), high-resolution transmission electron microscopy (HRTEM) and energy-dispersive X-ray (EDX) spectroscopy on a FEI TALOS F200X transmission electron microscope with a superX-G2 energy dispersive X-ray spectrometer. The Ce-UiO-66 were dissolved in a mixture of 10 % deuterochloric acid (DCl) in D_2O and deuterated dimethyl sulfoxide (d6-DMSO) (molar ratio 1:7) for ^1H and ^{13}C nuclear magnetic resonance (NMR) measurements recorded by Bruker Avance III-HD 500 spectrometer. The Fourier-transform infrared (FTIR) spectroscopy was used to analyze the FTIR spectrum of Ce-UiO-66 by bromide pellet method on a Fourier transform spectrometer (ThermoFisher Scientific iS50, USA). The nitrogen adsorption isotherms were recorded using a surface area and pore size analyzer (Micromeritics ASAP 2460), and the specific surface area and the pore size distribution were calculated from the adsorption data using the Brunauer-Emmett-Teller (BET) method and density functional theory (DFT) method, respectively, after degassing at 100 °C under vacuum for 12 h. The surface chemical composition of Ce-UiO-66 was measured using X-ray photoelectron spectroscopy (XPS) on an Escalab 250Xi X-ray photoelectron spectromater (ThermoFisher Scientific, USA). The particle sizes and zeta potentials of the nanoparticles in water were measured using a Zetasizer Pro nanometer size and potential analyzer (Malvern Panalytical, UK) at 25 ℃ for three times. The ultraviolet-visible (UV-Vis) absorption was recorded an UV-3600 UV-Vis spectrophotometer (Shimadzu, Japan).

Extraction of mesenchymal stem cell membrane. The mesenchymal stem cells were isolated from the femur and tibia of Sprague-Dawley (SD) rats and cultured in DMEM/F12 medium (GIBCO, USA) supplemented with 10% fetal bovine serum (FBS, Gibco, USA) at 37 °C with 5% CO_2. To harvest the cell membranes, mesenchymal stem cells were separated using 0.25% trypsin-EDTA and centrifuged at 1000×*g* for 5 min. The cells were resuspended in a low-permeability lysis buffer at 4°C and homogenized with an ultrasonic disruptor for 30 min. The resulting supernatant was collected after centrifugation at 2000×*g* for 30 min at 4 °C followed by another round of centrifugation at 20,000×*g* for 60 min at 4 °C. The pellet (containing the isolated cell membrane) was stored at -20 °C for subsequent experiments. The concentration of the membrane protein from the mesenchymal stem cells was determined using a bicinchonininc acid (BCA) protein assay kit (ThermoFisher Scientific, USA).

Preparation of Ce-UiO-CM. Mesenchymal stem cell membrane with a final protein concentration of 0.2 mg·mL^{-1} was added to a dispersion of 0.25 mg·mL^{-1} Ce-UiO-66, and the mixture was sonicated at 42 kHz in water bath for 2 min. The mixed solution was then centrifuged at 20,000×*g* for 10 min at 4 °C, and the resulting pellet was collected to obtain the mesenchymal stem cell membrane-coated Ce-UiO-66 (referred to as Ce-UiO-CM). The concentration of the membrane protein in Ce-UiO-CM was determined by BCA method. ICG-modified Ce-UiO-CM was prepared by encapsulating the cell membrane on the ICG-modified Ce-UiO-66.

Catalase (CAT) activity. To detect the CAT-like activity of Ce-UiO-CM, Ce-UiO-CM (0.02 mg·mL^{-1}) was added into 10 mL H_2O_2 solution (100 μmol L^{-1}). The oxygen concentration in the solution was continuously monitored for 5 min using a JPSJ-605F dissolved oxygen meter (INESA, China). 100 μmol L^{-1} separate H_2O_2 solution and H_2O_2 adding with Ce-UiO-66 and CM of the corresponding concentrations were set as the control.

Identification of cell surface CD44, and CD45 on Ce-UiO-CM. The separate Ce-UiO-CM and Ce-UiO-CM treated with 10% fetal bovine serum (FBS) were incubated with anti-CD44 and anti-CD45 antibodies at room temperature in dark for 20 minutes, and detected by flow cytometry (BD Biosciences, LSR Fortessa X20, USA).

Western Blot Assay. The surface marker CD18 on Ce-UiO-66, CM, Ce-UiO-CM and Ce-UiO-CM treated with 10% FBS for 24h were performed by Western blotting assay. The protein was separated in 10% SDS-PAGE and electrophoretically transferred to polyvinylidene fluoride (PVDF) membrane, followed by blocking with 5% nonfat milk in TBS-T (Tris-HCl 20 mM, NaCl 136 mM, Tween-20 0.01%) for 1 h at room temperature. The membrane was then incubated overnight at 4 °C with the antibodies against CD18, followed

by incubation with Anti-Rabbit secondary antibody at room temperature for 1 h. The specific bands were observed using an odyssey infrared imaging system (LI-COR, CLx, USA).

***In vitro* thrombolysis treatment.** Blood clot samples prepared from fresh mouse blood were treated with saline, Ce-UiO-66, Ce-UiO-CM, ultrasound (US), Ce-UiO-66 + US, or Ce-UiO-CM + US. The working frequency of the US was 3 MHz, the duty ratio was 20%, the sound intensity was 1.4 $W \cdot cm^{-2}$, and the treatment time was 30 min. The aqueous dispersions of Ce-UiO-66 and Ce-UiO-CM with equivalent Ce-UiO-66 concentration (0.2 $mg \cdot mL^{-1}$) were prepared. The hemoglobin level in different treatment groups was assessed by measuring the absorbance of the supernatant at 540 nm using a Synergy H1 microplate reader (BioTek, USA). The weight changes of the blood clots before and after treatment in different treatment groups were recorded to evaluate the *in vitro* thrombolytic efficiency. All experiments were repeated for 4 times.

Cells and animals. Human umbilical vein endothelial cells (HUVECs) used in this study were purchased from the Cell Bank of the Type Culture Collection of the Chinese Academy of Sciences (Shanghai, China). Male SD rats (6-8 weeks, 180-200g) were purchased from Shanghai Shengchang Biotechnology Co., Ltd. All experimental operations were carried out in accordance with the approved guidelines of Ren Ji Hospital Ethics Committee.

Cell viability. HUVECs (100 µL suspension) were seeded into a 96-well plate at the density of 1×10^4 cells/well and cultured at 37 °C with 5% CO_2 for 24 h. These cells were treated with ultrasound (working frequency 3 MHz, duty ratio 20%) at different sound intensities (1.2, 1.3, 1.4, 1.5, 1.6, 1.7 $W \cdot cm^{-2}$) for 10 min, with 4 replicates for each power level. The effect of different ultrasound intensities on the viability of HUVECs was studied with the CCK-8 kit (DOJINDO, Japan), and the absorbance was measured using a Synergy H1 microplate reader. The aqueous dispersions of Ce-UiO-66 and Ce-UiO-CM with different equivalent Ce-UiO-66 concentrations (0.0001, 0.0002, 0.001, 0.002, 0.01, 0.02 $mg \cdot mL^{-1}$) were used to study the effects of Ce-UiO-66 and Ce-UiO-CM on the viability of HUVECs. HUVECs cultured in DMEM only were used as control. For combinatory treatment with nanoenzyme and ultrasound, cells were additionally stimulated with ultrasound under a working frequency of 3 MHz, duty ratio of 20%, and sound intensity of 1.4 W cm^{-2} for 10 min. 4 replicates were set up for each treatment condition. The cell viability was calculated according to the instruction of the CCK-8 kit.

Cellular antioxidant enzyme activity. The 2',7'-dichlorodihydrofluorescein diacetate (DCFH-DA) assay kit (Beyotime, China) was used to detect intracellular ROS. HUVECs were cultured in a confocal culture dish for 24 h before being incubated with H_2O_2 (100 µmol

L^{-1}) or H_2O_2 (100 μmol L^{-1}) + Ce-UiO-CM (0.02 mg·mL^{-1}) for 4 h. Untreated HUVECs were used as negative control. For intracellular ROS detection, cells were incubated with 10 μmol L^{-1} DCFH-DA (Beyotime, China) for 30 min, followed by 4% paraformaldehyde for 20 min and 10 μg·mL^{-1} 4',6-diamidino-2-phenylindole (DAPI, Beyotime, China) for 5 min. The 2',7'-dichlorofluorescein (DCF) fluorescence in the cells was observed under a FV 3000 confocal laser scanning microscopy (Olympus, Japan; excitation wavelength: 488 nm for DCF, 405 nm for DAPI; emission wavelength: 500 nm-600 nm for DCF, 420 nm-480 nm for DAPI) to evaluate the intracellular ROS level.

Establishment of the rat femoral artery thrombosis model using the $FeCl_3$ injury method. SD rats were anesthetized, and their hind limbs were immobilized. A longitudinal incision was made on the medial aspect of the thigh skin, and the muscles were carefully separated to expose the femoral artery. The femoral artery was fully wrapped by a piece of filter paper (3 mm × 5 mm) pre-soaked in 5% $FeCl_3$ solution. A plastic wrap was used to cover the filter paper to prevent damage to other tissues caused by $FeCl_3$. After 1 min, the plastic wrap and the filter paper were removed, and the wound area was gently rinsed with saline. The incision was closed again with sutures and the rats were monitored until fully awake.

Fluorescence imaging. To evaluate the *in vivo* distribution of ICG-modified Ce-UiO-CM, the thrombosed rats were injected with 100 μL of ICG-modified Ce-UiO-66 or Ce-UiO-CM (containing 1 mg·mL^{-1} of Ce-UiO-66) via the tail vein (n = 4 for each group). Fluorescence images of live animals were collected at different time points (0, 0.5, 1, 2, 3, 4, 6, 24 h) after injection using an AniView100 system (BLT, China). The rats were sacrificed at 0.5, 2 and 24 h after injection, and major organs including heart, liver, spleen, lung, kidney, as well as the femoral artery (thrombus site) were collected for fluorescence imaging using an InVivo ART-100 (VISQUE, Korea).

Pharmacokinetic study. Four rats (4 males; weight 200 ± 10 g) were used to study the pharmacokinetic profiles of Ce-UiO-CM, which were injected with Ce-UiO-CM solution through tail vein injection (dose of Ce: 937.8 μg kg^{-1}) after being fasted for 12 h. Four rats Blood samples (200 μL) were collected from the ocular vein of each rat at 0.25, 1, 2, 4, 24, 48, and 72 h after intravenous administration, and the concentration of Ce was determined by inductively coupled plasma mass spectrometry (ICP-MS, Agilent 720ES, USA). The pharmacokinetic parameters including the area under the concentration-time curve (AUC)

from zero to the last measurable plasma concentration point (AUC_{0-t}), AUC from 0 to infinity ($AUC_{0-\infty}$), mean residence time ($MRT_{0-\infty}$), terminal elimination half-life ($T_{1/2}$) and clearance (CL) were calculated by a non compartment model using MaS Studio 1.6.0.5 software.

Photoacoustic imaging. To verify the *in vivo* oxygen production ability of Ce-UiO-CM, the thrombosed rats were injected with 100 μL of Ce-UiO-CM (containing 1 $mg \cdot mL^{-1}$ of Ce-Uio-66) via the tail vein, and photoacoustic images of the femoral artery area were collected at different time points (0, 0.5, 1, 1.5, 2, 4 h) using Vevo 2100 LAZR and VEVO LAZR-X Imaging System (Visual Sonic, Canada) to determine the blood oxygen saturation (sO_2). The thrombosed rats injected with 100 μL of Ce-UiO-66 and CM at corresponding concentrations were treated as comparison group. The sO_2 was quantitatively analyzed by Vevo LAB version 5.5.1.

***In vivo* thrombolysis treatment.** To evaluate the therapeutic effect of Ce-UiO-CM in thrombolysis treatment *in vivo*, the thrombosed rats were randomly divided into six treatment groups (*n* = 5 in each group): saline group, Ce-UiO-66 group, Ce-UiO-CM group, ultrasound (US) group, Ce-UiO-66 + US group, and Ce-UiO-CM + US group. Ultrasound with the working frequency of 3 MHz, duty ratio of 20%, and sound intensity of 1.4 $W \cdot cm^{-2}$ was used for US treatment. 100 μL aqueous dispersion of Ce-UiO-66 or Ce-UiO-CM with equivalent Ce-UiO-66 concentration (1$mg \cdot mL^{-1}$) were injected via the tail vein 0.5 h before US treatment (10 min for 3 times). Doppler ultrasound imaging was used to detect changes in the blood flow at the site of femoral artery occlusion through a Vevo 2100 LAZR Imaging System. The femoral artery was collected for hematoxylin-eosin (H&E) and Masson's trichrome staining after treatment, and the stained sections were observed using an optical microscope. Additionally, the femoral artery was also stained with dihydroethidium (DHE) and α-SMA antibody for fluorescence analysis. The femoral artery of the thrombus model and normal animal were stained with intercellular adhesion molecule 1 (ICAM-1) antibody. Image J software was used to evaluate the treatment effect, calculated as follows: percentage of thrombus area = area of thrombus/total area of femoral artery × 100%.

Safety evaluation. The rats treated with the above anti-thrombotic therapy were sacrificed, and blood samples were collected from the orbital socket for routine blood test, as well as analysis of serum biochemical parameters associated with liver, renal and cardiac functions. The major organs such as heart, liver, spleen, lung, and kidney were collected for H&E staining to evaluate the organ toxicity of the treatment.

Statistical analysis. All the statistical values in this study were displayed as mean ± standard error of mean (SEM) for $n \geq 3$ independent experiments. Student's t-test was used to compare the statistical differences between two independent groups. Comparisons among multiple groups were analyzed by one-way analysis of variance (ANOVA). All statistical analyses were conducted with GraphPad Prism software (PRISM 9.0). $P < 0.05$ was regarded as statistically significant.

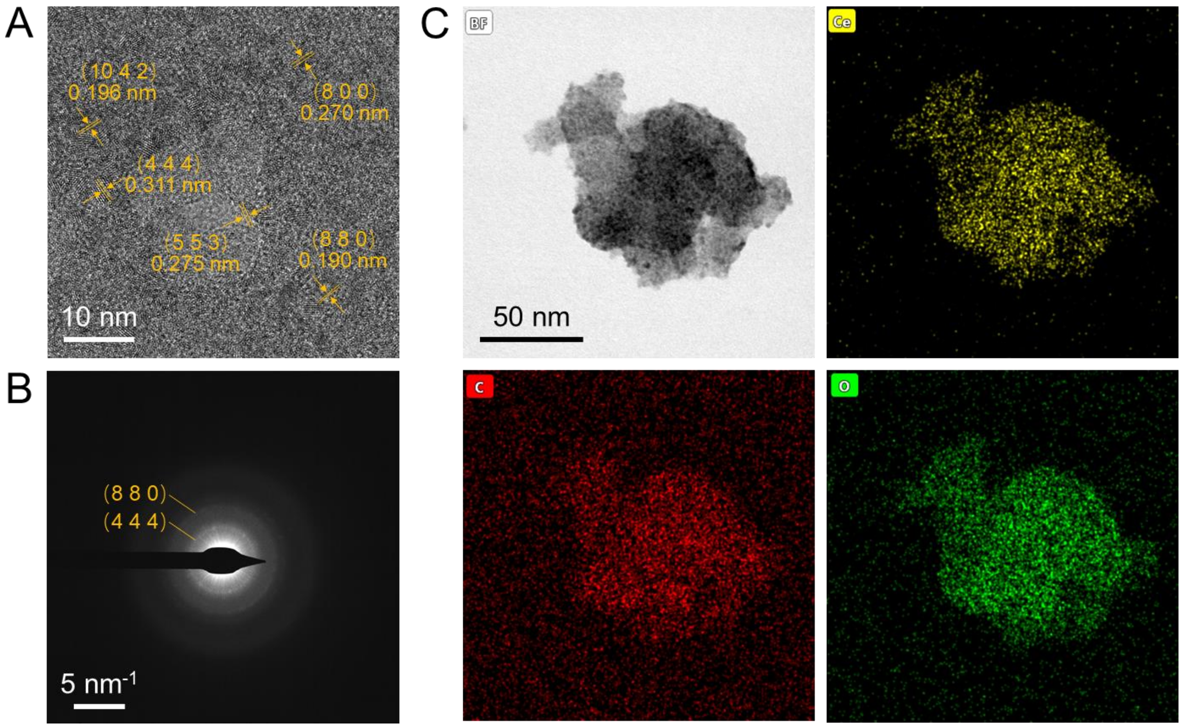

Figure S1. The structural characterization of Ce-UiO-66.

The HRTEM image (A), selected area electron diffraction image (B) and energy-dispersive X-ray (EDX) spectroscopy elemental mapping (C) of Ce-UiO-66.

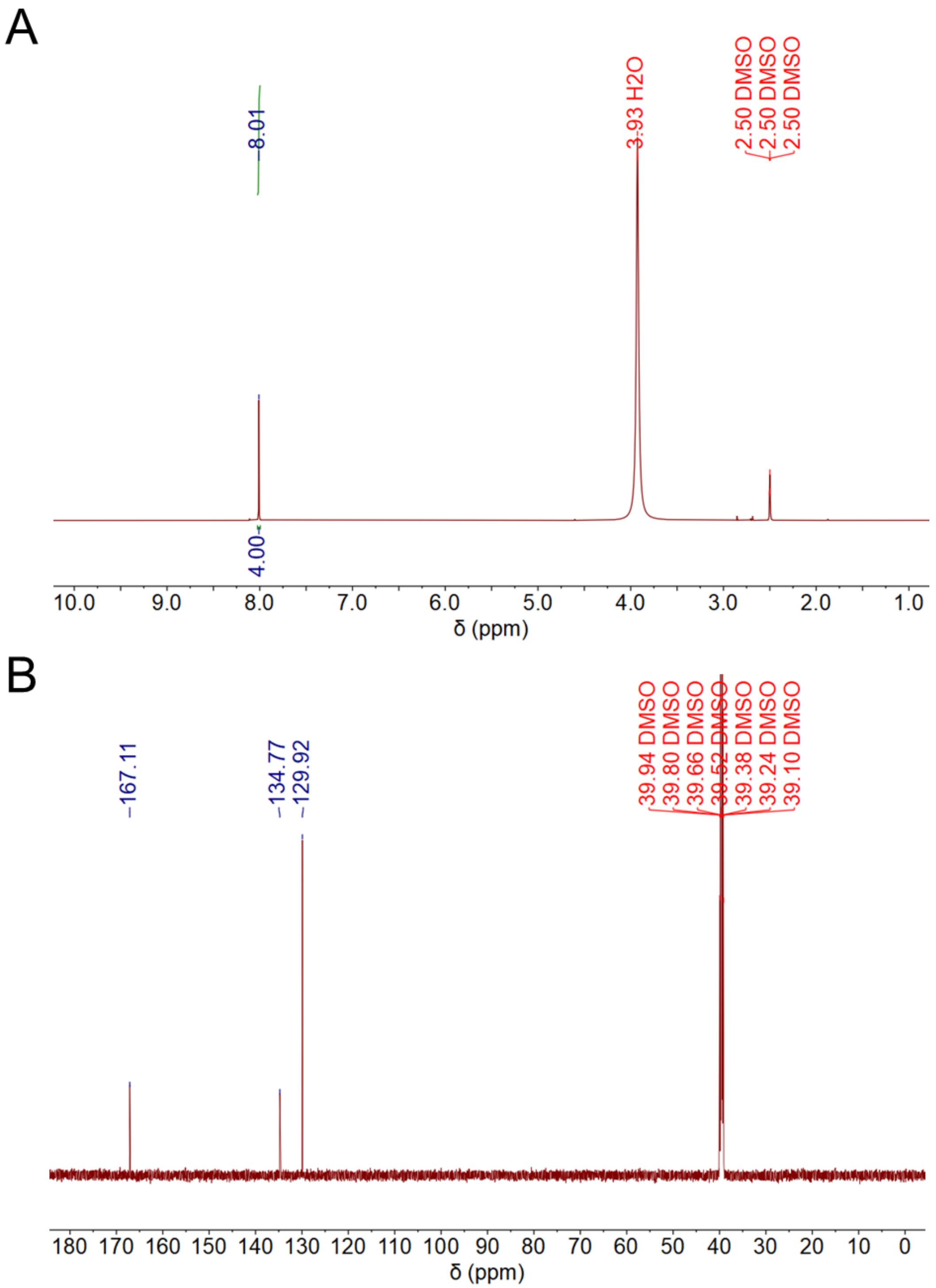

Figure S2. The NMR spectra of Ce-UiO-66.

A) ^{1}H NMR spectrum of dissolved Ce-UiO-66. ^{1}H NMR (600 MHz, DMSO-d_6): δ = 8.01 (s, 4H, H_2BDC) ppm. B) ^{13}C NMR spectrum of dissolved Ce-UiO-66. ^{13}C NMR (151 MHz, DMSO-*d*6): δ = 167.11, 134.77, 129.92 ppm.

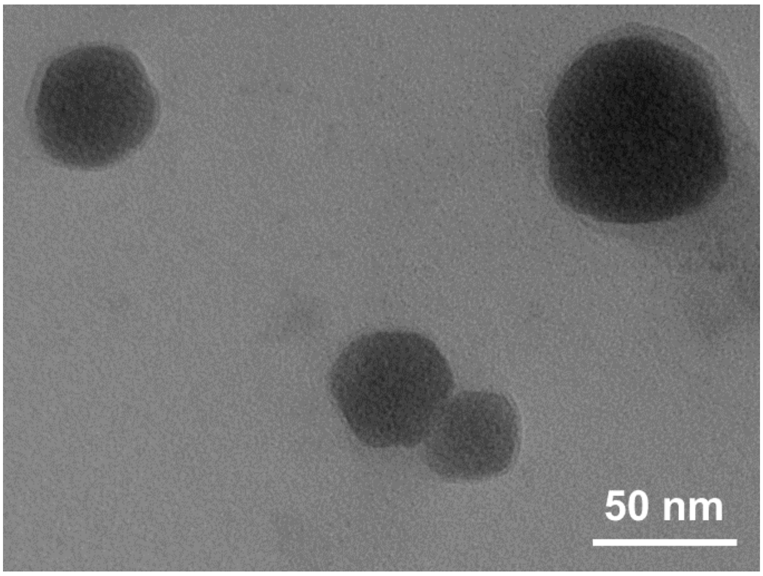

Figure S3. The TEM image of Ce-UiO-CM.

The obtained Ce-UiO-CM exhibited a core-shell structure with a layer of membrane on the surface. Scale bar is 50 nm.

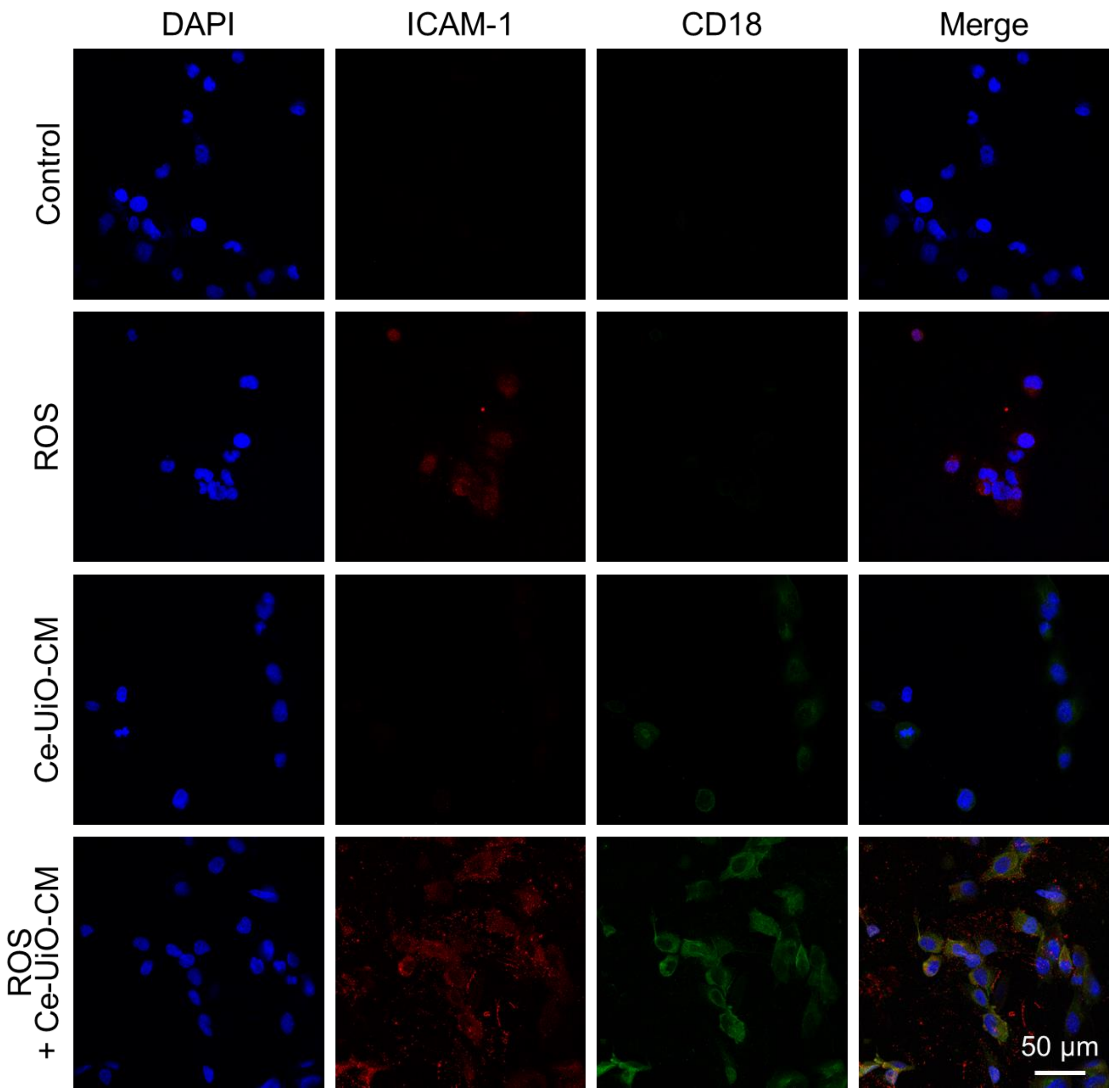

Figure S4. The cellular uptake of the Ce-UiO-CM in HUVECs.

The HUVECs were pretreated with or without ROS for 0.5 h, and then incubated with Ce-UiO-CM for 4 h. After ROS treatment, the intercellular adhesion molecule 1 (ICAM-1) expressions were increased on HUVECs. Additionally, intense green fluorescence from CD18 on Ce-UiO-CM was notably evident in HUVECs exhibiting high ICAM-1 expression after 4 hours of incubation with Ce-UiO-CM, whereas HUVECs with low ICAM-1 expression only gave the faint fluorescence. These findings suggest enhanced cellular uptake of Ce-UiO-CM by HUVECs with elevated ICAM-1 levels, highlighting the specificity of Ce-UiO-CM for targeting inflamed endothelial cells. Scale bar is 50 μm.

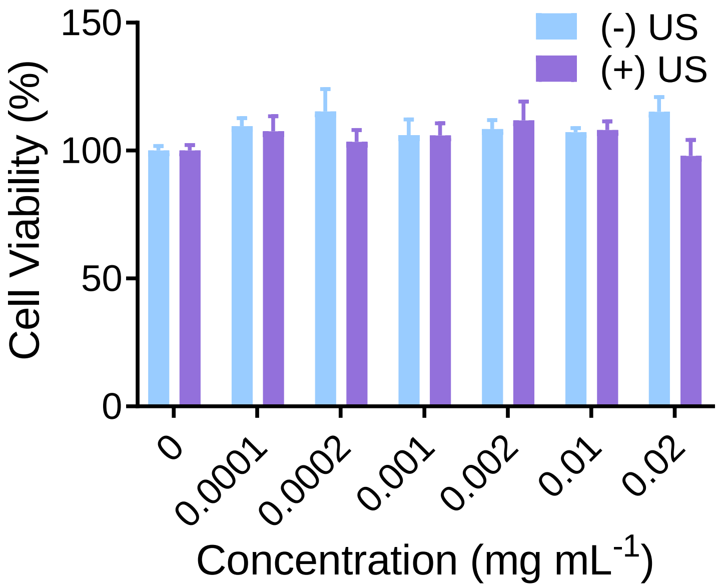

Figure S5. *In vitro* cytotoxicity of different concentrations of Ce-UiO-66 with or without ultrasound treatment.

HUVECs were treated with Ce-UiO-66 (0, 0.0001, 0.0002, 0.001, 0.002, 0.01 or 0.02 mg·mL^{-1}) and then treated with (+) or without (-) ultrasound stimulation. Cell viability was calculated according to CCK-8 assay. Data are presented as mean ± standard error of mean (SEM), n = 4. One-way analysis of variance (ANOVA) with Tukey's test for multiple comparisons. No obvious changes were observed.

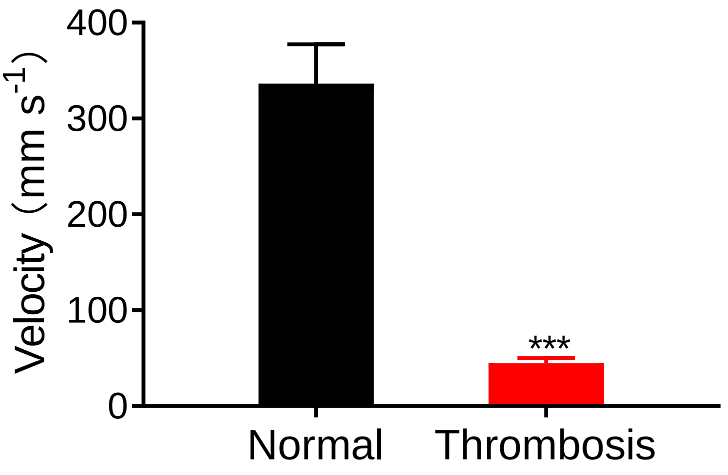

Figure S6. The ultrasonic blood flow analysis of the femoral artery thrombosis model. Data are presented as mean ± standard error of mean (SEM), n = 5. $^{***}P < 0.001$, student's t-test. The blood flow in the injured vessel was significantly reduced after thrombus formation.

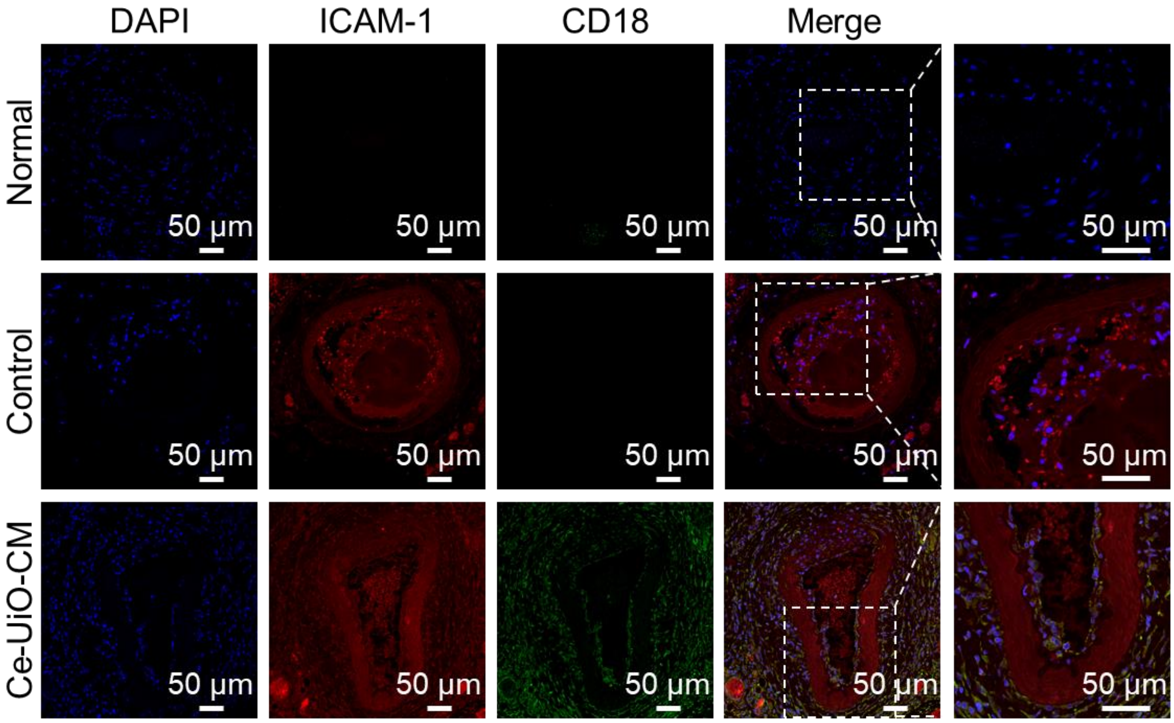

Figure S7. ***In vivo*** **targeting of Ce-UiO-CM for inflammatory endothelial cells at the site of thrombosis.**

Elevated expression of Intercellular Adhesion Molecule-1 (ICAM-1) was observed on the vascular endothelium within the thrombosis region compared to the normal vascular tissue. Pronounced green fluorescence emanating from CD18 on Ce-UiO-CM was predominantly localized in the vascular endothelium exhibiting ICAM-1 overexpression, indicating the specificity of Ce-UiO-CM for targeting inflammatory endothelial cells in the thrombotic area. Scale bar is 50 μm.

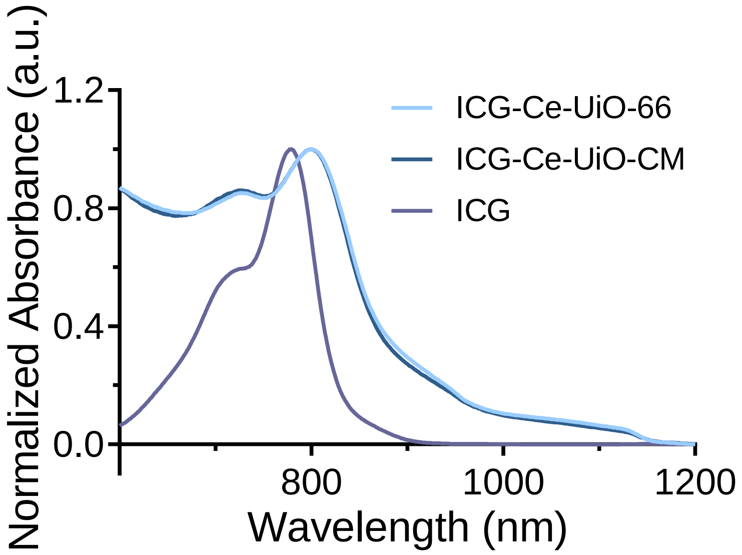

Figure S8. The ultraviolet-visible (UV-Vis) absorption spectra of ICG-modified Ce-UiO-CM and Ce-UiO-66 in water.

Compared with ICG (about 780nm), the absorption peaks of ICG-modified Ce-UiO-CM and Ce-UiO-66 had a slight red shift at about 800 nm, suggesting the successful assembly of ICG into Ce-UiO-66.

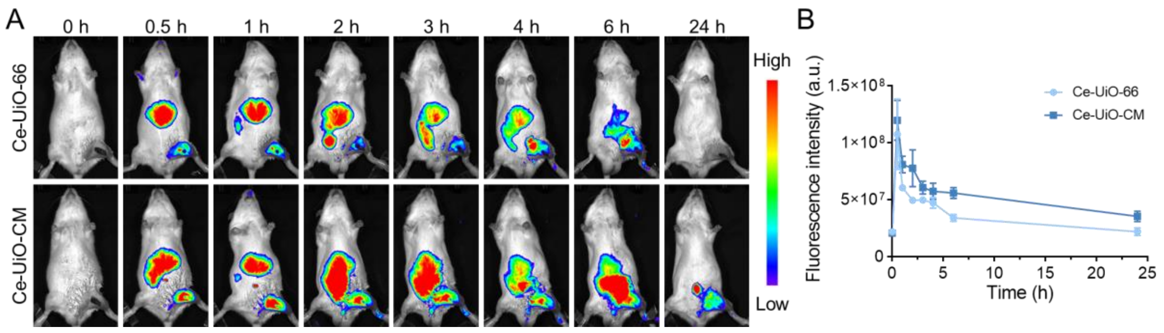

Figure S9. The fluorescence distribution of ICG-modified Ce-UiO-CM and Ce-UiO-66 *in vivo*.

A) The fluorescence images were collected at 0, 0.5, 1, 2, 3, 4, 6 and 24 h after Ce-UiO-CM or Ce-UiO-66 injection through the tail vein. The enlarged fluorescent images of the right leg thrombosis modeling area were presented in Figure 5D. B) The quantitative analysis of corresponding fluorescence intensity of thrombosis area in (A). Data are presented as mean ± standard error of mean (SEM), $n = 4$.

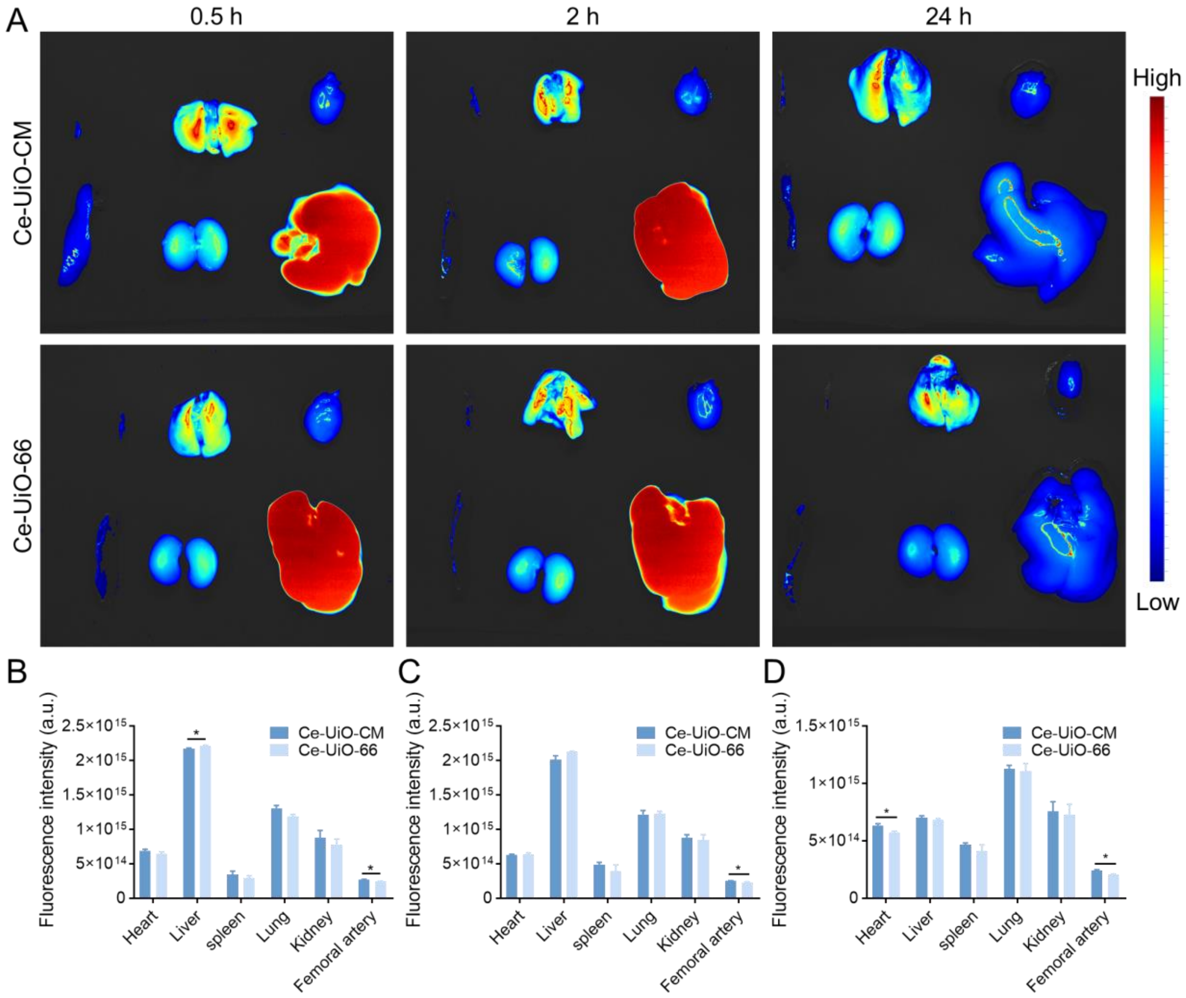

Figure S10. Distribution of ICG-modified Ce-UiO-CM and Ce-UiO-66 in the major organs and the femoral artery thrombus site at 0.5, 2 and 24 h after injection.
The fluorescence images (A) and quantitative analysis of the major organs and the femoral artery thrombus site after injection 0.5 h (B), 2 h (C) and 24 h (D) of ICG-modified Ce-UiO-CM and Ce-UiO-66. In (A), the first row showed the femoral artery, lungs, and heart from left to right, while the second row showed the spleen, kidneys, and liver from left to right. Data are presented as mean ± standard error of mean (SEM), $n = 4$. $^{*}P < 0.05$, student's t-test.

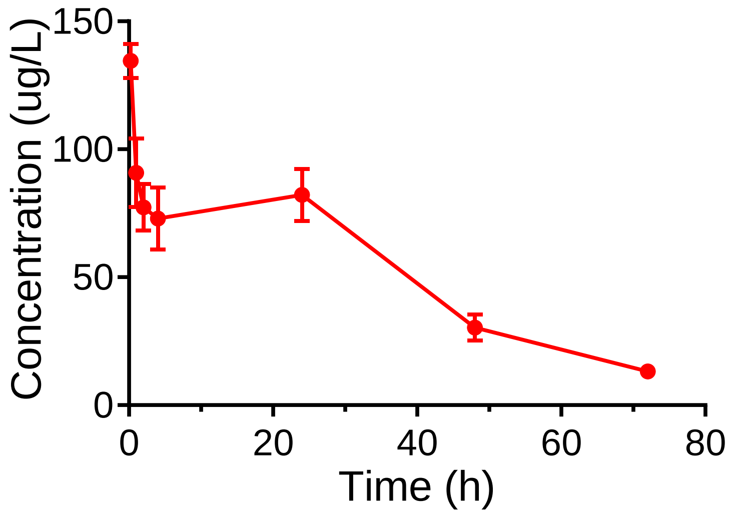

Figure S11. Mean plasma concentration-time curves of Ce-UiO-CM after a single intravenous administration at a dose of 937.8 μg kg^{-1} of Ce in healthy rats.
The concentration of Ce messured by inductively coupled plasma mass spectrometry (ICP-MS). Data are presented as mean ± standard error of mean (SEM), *n* = 4.

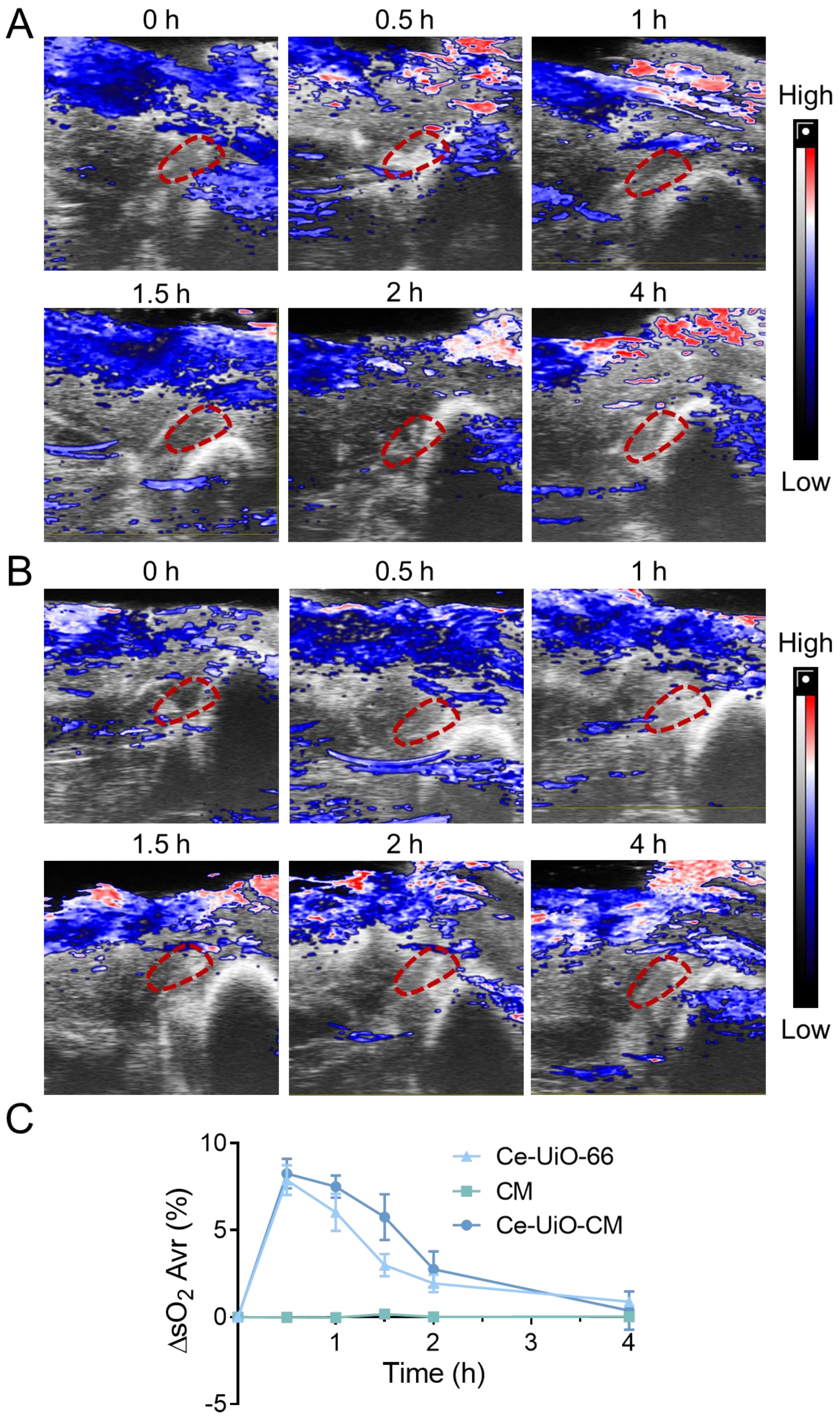

Figure S12. Evaluation of the oxygen production ability of Ce-UiO-66 and CM *in vivo*. The photoacoustic signal, indicative of the blood oxygen saturation level, at the embolic site after Ce-UiO-66 (A) and CM (B) administration. C) The increase of blood oxygen saturation level at the embolic site after Ce-UiO-66, CM and Ce-UiO-CM injection through the caudal vein. The ΔsO_2 of Ce-UiO-CM group was calculated according to Figure 6D. Data are presented as mean ± standard error of mean (SEM), *n* = 4.

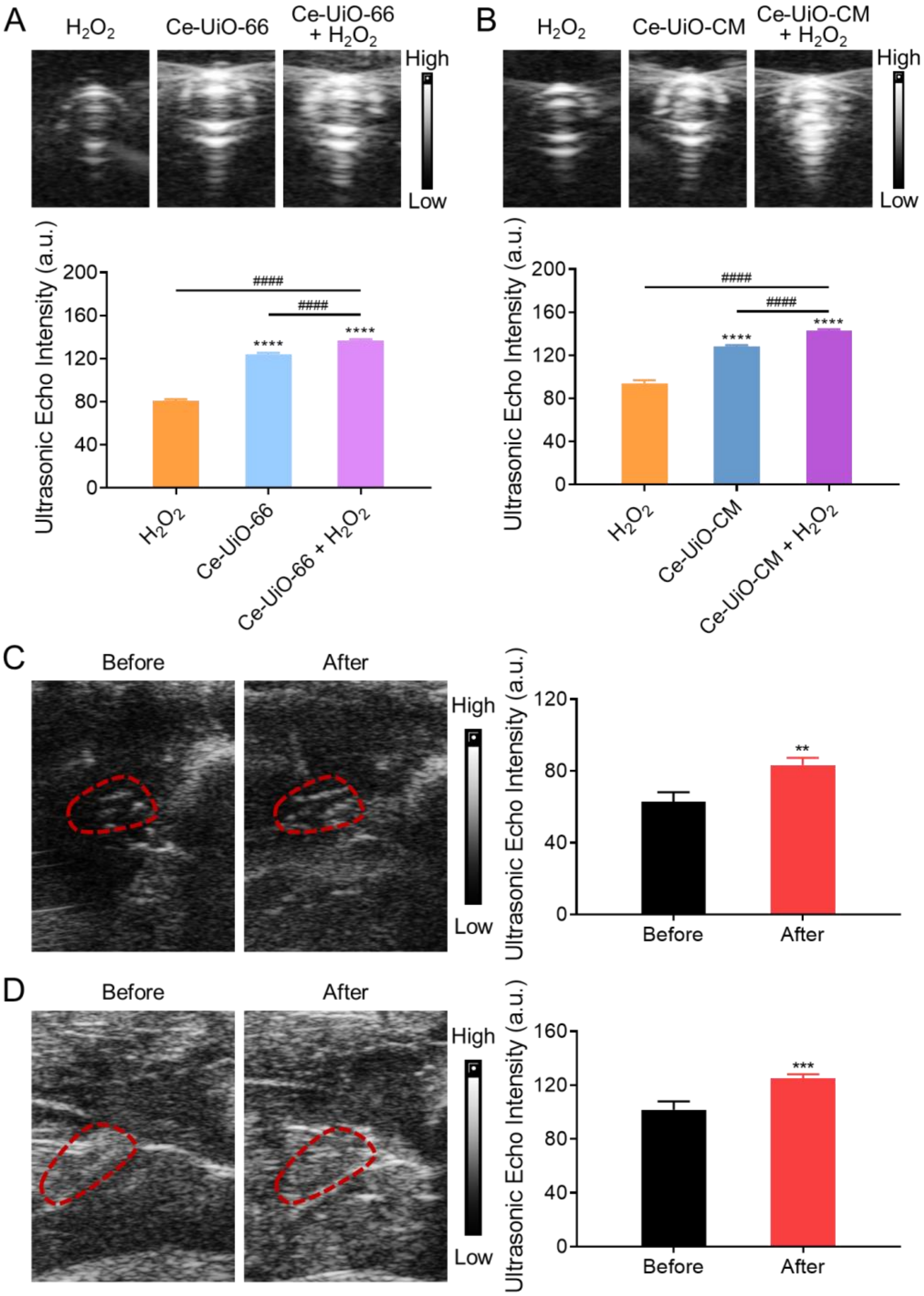

Figure S13. Ultrasound imaging of thrombotic areas after intravenous injection of Ce-UiO-CM in a femoral artery thrombi-bearing rat model.

A) Ultrasound images and ultrasound echo intensities of H_2O_2, Ce-UiO-66, and Ce-UiO-66 + H_2O_2, respectively. Data are presented as mean ± standard error of mean (SEM), n = 5. $^{\#\#\#\#}$, $^{****}P < 0.0001$, ANOVA with Tukey's multiple comparison. B) Ultrasound images and ultrasound echo intensities of H_2O_2, Ce-UiO-CM, and Ce-UiO-CM + H_2O_2, respectively. Data are presented as mean ± standard error of mean (SEM), n = 5. $^{\#\#\#\#}$, $^{****}P < 0.0001$, ANOVA with Tukey's multiple comparison. C) *In vivo* ultrasound images and the ultrasound echo intensities of femoral artery thrombi before and after Ce-UiO-66 injection in B mode.

Data are presented as mean ± standard error of mean (SEM), n = 5. $^{**}P < 0.01$, student's t-test. D) *In vivo* ultrasound images and the ultrasound echo intensities of femoral artery thrombi before and after Ce-UiO-CM injection in B mode. Data are presented as mean ± standard error of mean (SEM), n = 5. $^{***}P < 0.001$, student's t-test.

Upon the conversion of H_2O_2 to oxygen, the ultrasound echo intensities of Ce-UiO-66 + H_2O_2 group and Ce-UiO-CM + H_2O_2 group were significantly enhanced. After injection of Ce-UiO-66 and Ce-UiO-CM, the B-mode ultrasound echo intensity at the thrombus site was significantly enhanced.

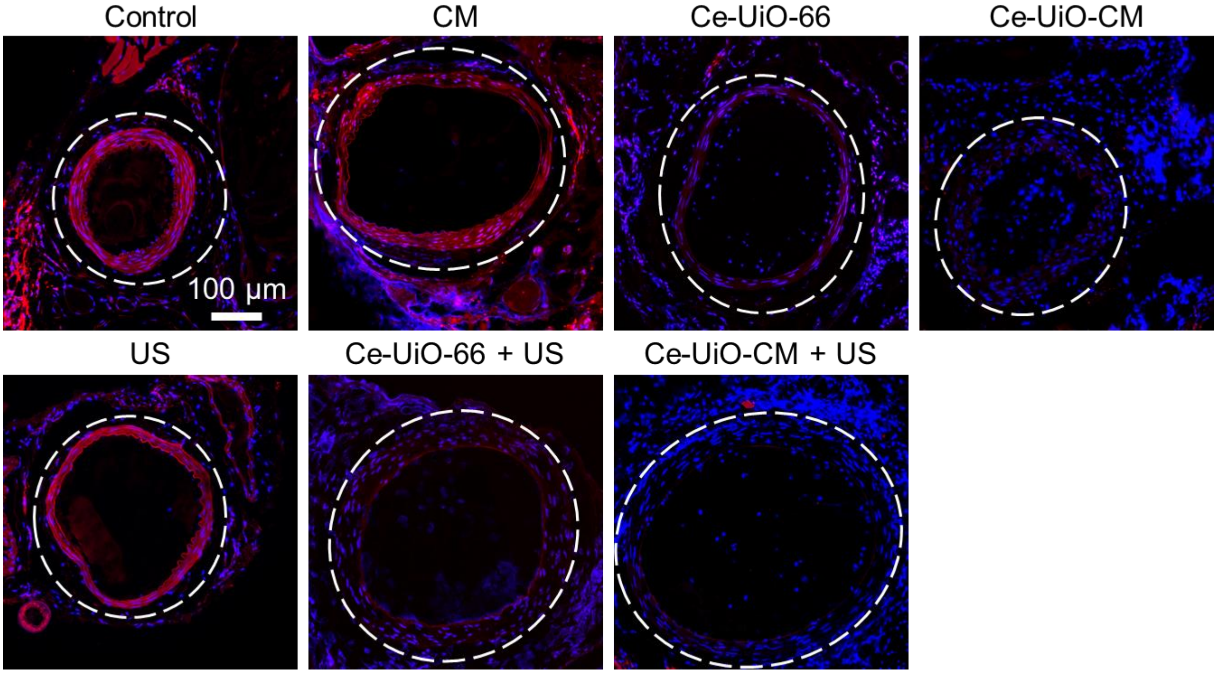

Figure S14. The fluorescence images of DHE-stained femoral artery in different treatment groups.

The white circle displays the thrombus modeling blood vessels. Scale bar is 100 μm. The endothelium of the femoral artery in the control, CM and US group showed a high intensity of DHE fluorescence while the fluorescence were significantly reduced after Ce-UiO-66 and Ce-UiO-CM treatment with or without US. Additionally, the red fluorescence of DHE in these Ce-UiO-CM treated groups were weaker than these Ce-UiO-66 treated groups.

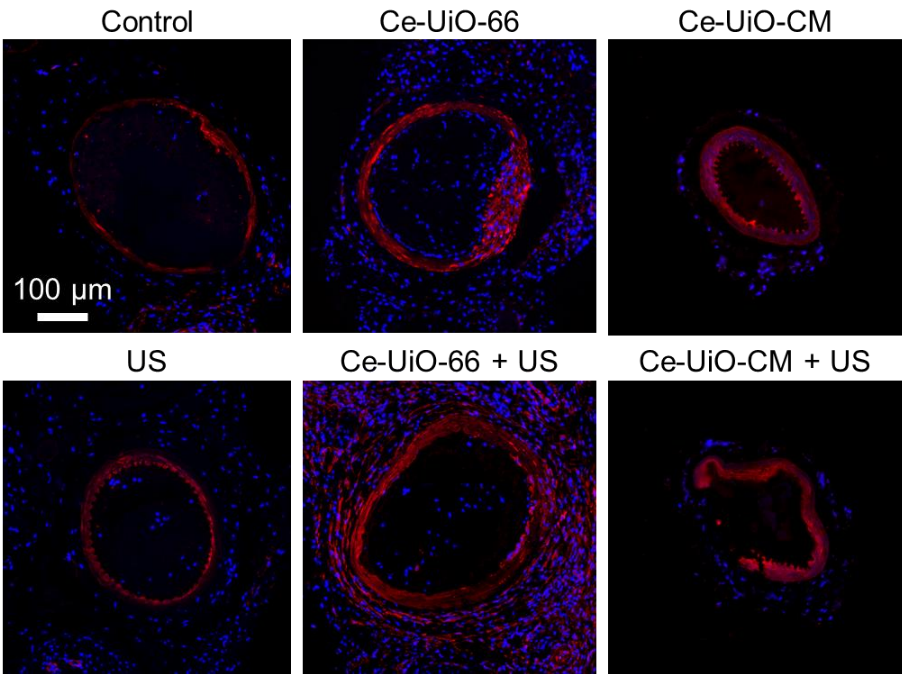

Figure S15. Immunofluorescence iamges of the femoral artery in different treatment groups stainied with smooth muscle cell marker α-smooth muscle actin (α-SMA). The number of VSMCs were significantly increased in the thrombotic area after treating with Ce-UiO-66 and Ce-UiO-CM. Scale bar is 100 μm.

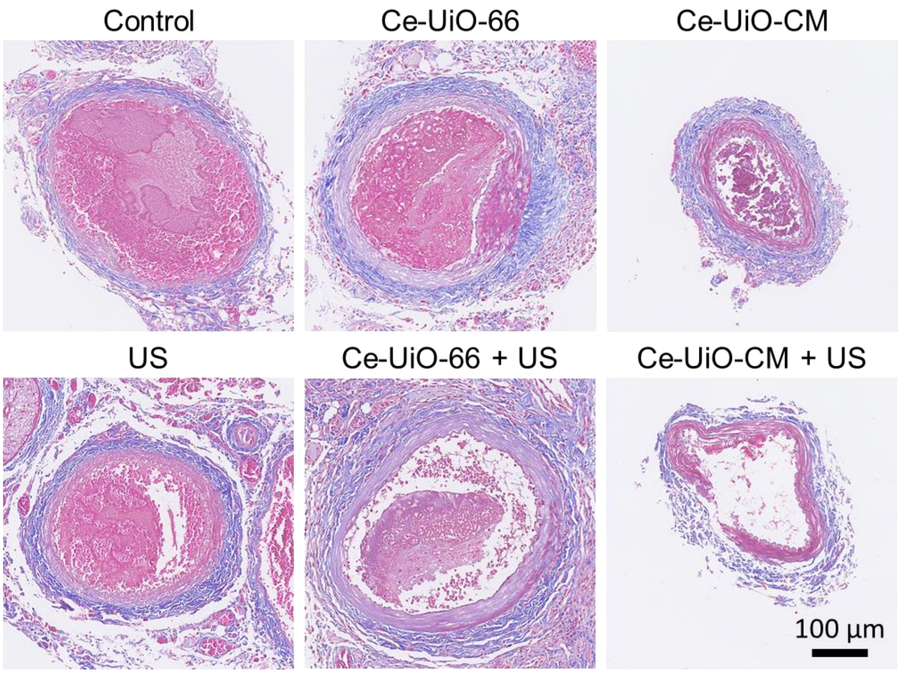

Figure S16. Masson's trichrome staining diagram of the femoral artery in different treatment groups.

The concentration of collagen was also greatly upregulated after Ce-UiO-66 and Ce-UiO-CM treatment with or without US. Scale bar is 100 μm.

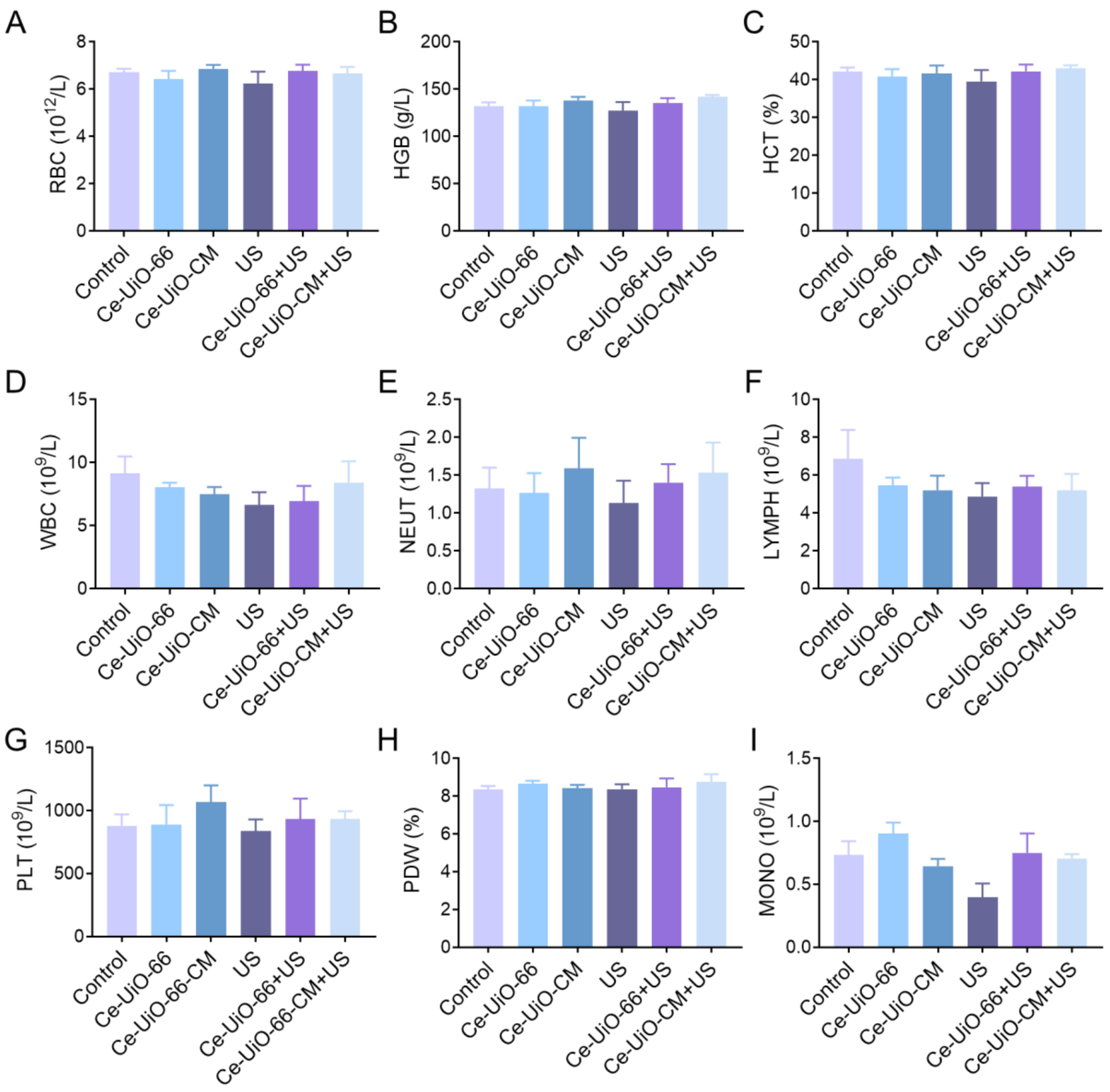

Figure S17. The routine blood test results of different treatment groups.

The levels of red blood cell count (RBC, **A**), hemoglobin (HGB, **B**), hematocrit (HCT, **C**), white blood cell count (WBC, **D**), neutrophil count (NEUT, **E**), lymphocyte count (LYMPH, **F**), platelet count (PLT, **G**), platelet volume distribution width (PDW, **H**), and monocyte count (MONO, **I**) in the blood samples from the treated rats were measured. Data are presented as mean ± standard error of mean (SEM), *n* = 4. ANOVA with Tukey's test for multiple comparisons. No obvious changes were observed.

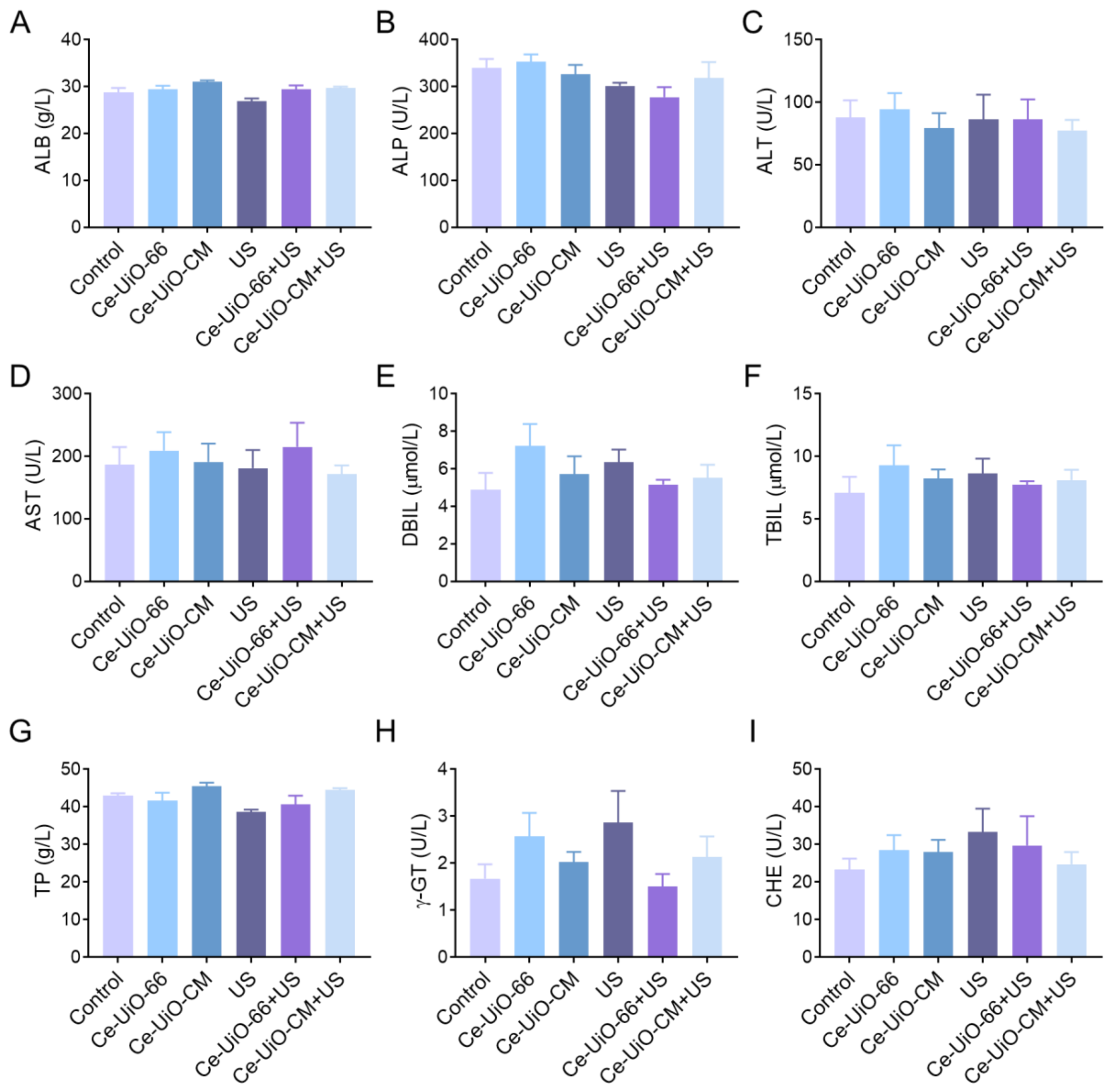

Figure S18. The liver function related serum biochemical parameters of different treatment groups.

The levels of albumin (ALB, **A**), alkaline phosphatase (ALP, **B**), alanine transaminase (ALT, **C**), aspartate transaminase (AST, **D**), direct bilirubin (DBIL, **E**), total bilirubin (TBIL, **F**), total protein (TP, **G**), γ-glutamyl transpeptidase (γ-GT, **H**), and cholinesterase (CHE, **I**) in the blood samples of the treated rats were measured. Data are presented as mean ± standard error of mean (SEM), n = 4. ANOVA with Tukey's test for multiple comparisons. No obvious changes were observed.

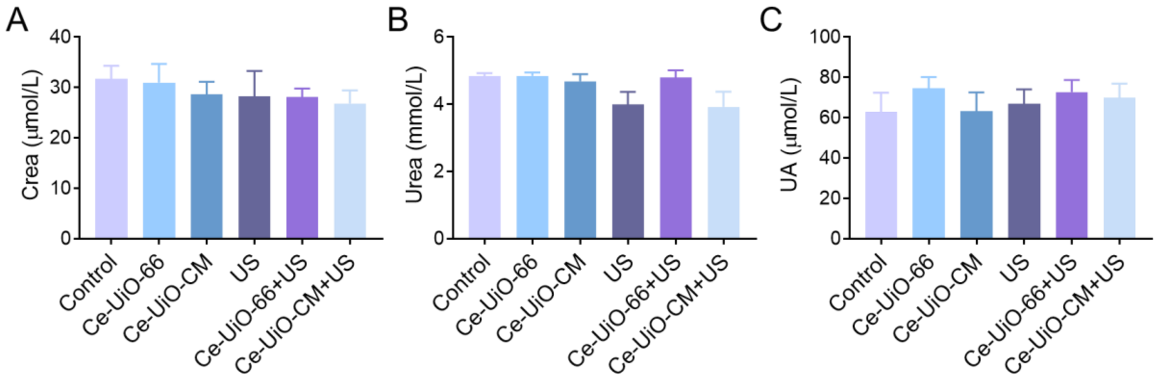

Figure S19. The renal function related serum biochemical parameters of different treatment groups.

The levels of creatinine (Crea, **A**), urea (Urea, **B**), and uric acid (UA, **C**) in the blood samples of the treated rats were measured. Data are presented as mean ± standard error of mean (SEM), n = 4. ANOVA with Tukey's test for multiple comparisons. No obvious changes were observed.

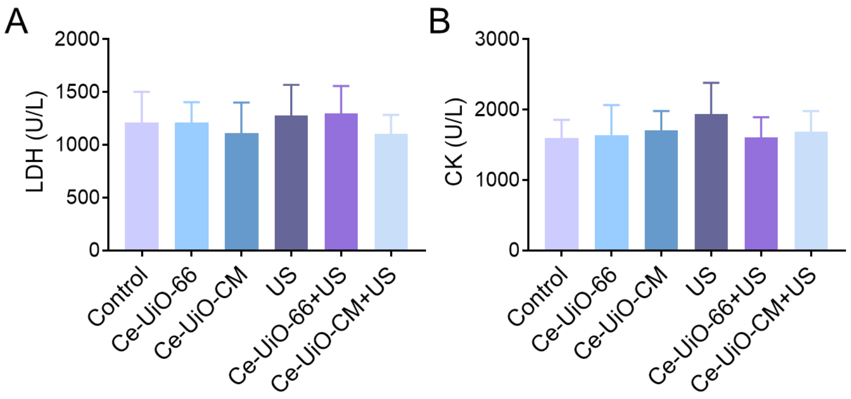

Figure S20. The cardiac function related serum biochemical parameters of different treatment groups.

The levels of lactate dehydrogenase (LDH, **A**) and creatine kinase (CK, **B**) in the blood samples of the treated rats were measured. Data are presented as mean ± standard error of mean (SEM), n = 4. ANOVA with Tukey's test for multiple comparisons. No obvious changes were observed.

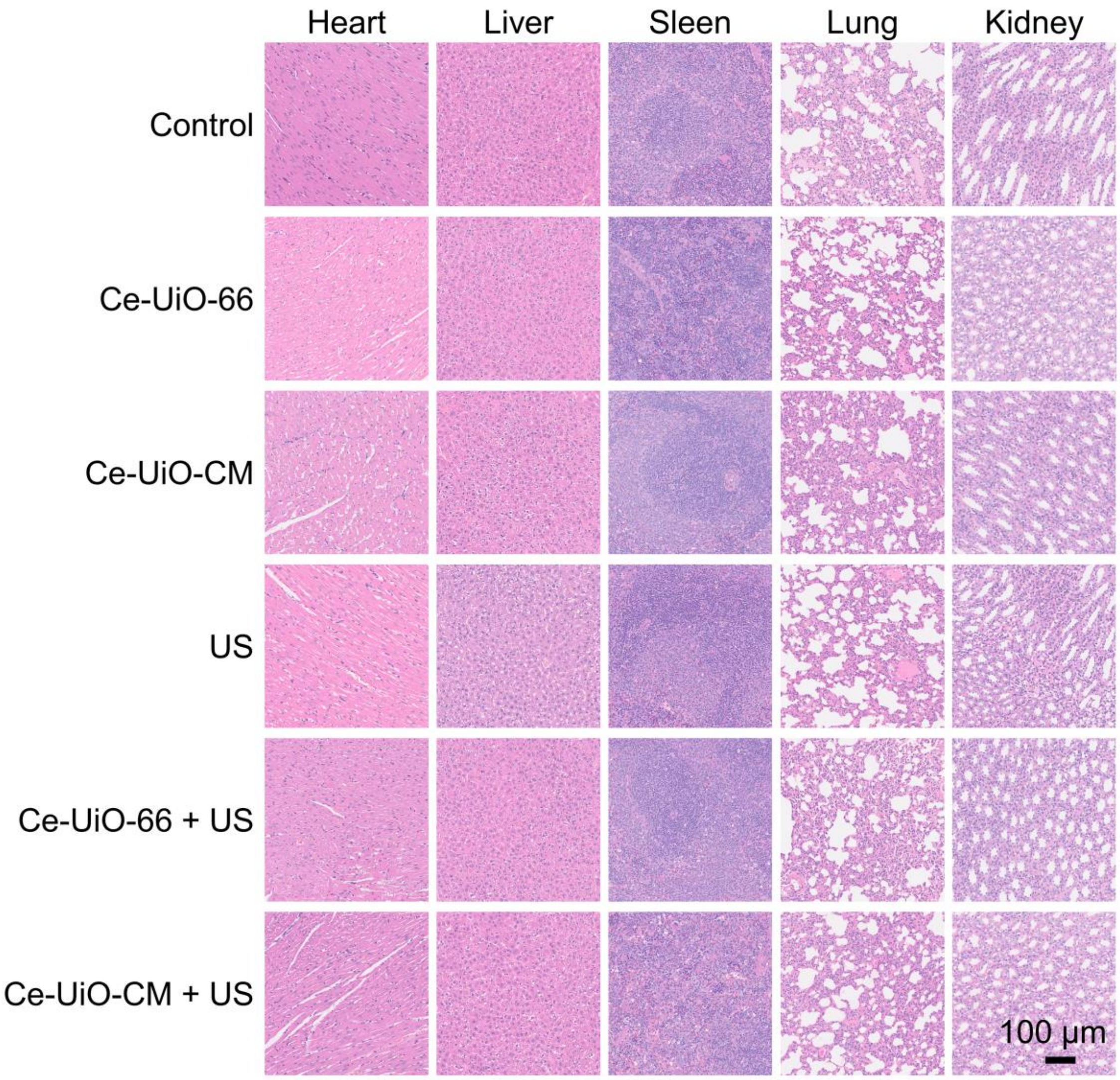

Figure S21. H&E staining of the histological sections of the major organs in different treatment groups.

The major organs (heart, liver, spleen, kidney and lung) of the thrombosed rats were sectioned and H&E stained after treatment. Scale bar is 100 μm. No obvious changes were observed.

Table S1. Assignment of the Ce $3d_{3/2}$, $3d_{5/2}$ components from the XPS spectra collected for Ce-UiO-66.

Ionic state	Spin-orbit doublet	Components	BE (±0.1 eV)	FWHM (±0.1 eV)	Ce(III) ratio (%)[a]
Ce(IV)	$3d_{3/2}$	u	901.7	2.6	35.0
		u^{II}	907.2	4.6	
		u^{III}	917.0	3	
	$3d_{5/2}$	v	883.2	2.5	
		v^{II}	888.6	4.6	
		v^{III}	898.5	2.9	
Ce(III)	$3d_{3/2}$	u^0	899.7	2.6	
		u^I	904.0	3.2	
	$3d_{5/2}$	v^0	881.4	2.6	
		v^I	885.7	3.2	

[a] The amount of Ce (III) and Ce(IV) was calculated from the peak area of the Ce 3d components using the following equations:

$Ce(IV) = u + u^{II} + u^{III} + v + v^{II} + v^{III}$

$Ce(III) = u^0 + u^I + v^0 + v^I$

Ce (III) ratio (%) = [Ce(III)/(Ce(IV)+Ce(III))]×100

Table S2. Assignment of the O 1s components from the XPS spectra collected for Ce-UiO-66.

Components	BE (±0.1 eV)	FWHM (±0.1 eV)	Percentage ratio (%)
O-C=O	533.2	1.5	26.4%
Ce-O-C	532.0	1.5	28.2%
Ce-OH	531.3	1.5	33.9%
Ce-O-Ce	529.8	1.5	11.5%

Table S3. Pharmacokinetic parameters of Ce-UiO-CM after a single intravenous administration at a dose of 937.8 μg kg^{-1} of Ce in healthy rats (n = 4).

Pharmacokinetic parameters	Unit	Values (mean ± SD)
AUC_{0-t}	μg L^{-1} h	3778.60 ± 569.99
$AUC_{0-\infty}$	μg L^{-1} h	4178.48 ± 434.25
$MRT_{0-\infty}$	h	32.67 ± 6.99
$T_{1/2}$	h	20.25 ± 4.53
CL	L h^{-1} kg^{-1}	0.23 ± 0.02
